# Supplementary material for: Protective effect of LNA-anti-miR-132 therapy on liver fibrosis in mice
Source: Mol Ther Nucleic Acids. 2021 May 14;25:155–67. doi: 10.1016/j.omtn.2021.05.007 (PMC8368790; doi:10.1016/j.omtn.2021.05.007)
Supplement: Document S1. Supplemental materials and methods, Figures S1–S5, and Table S2 [file mmc1.pdf]

OMTN, Volume *25*

Supplemental information

Protective effect of LNA-anti-miR-132 therapy on liver fibrosis in mice

Fatemeh Momen-Heravi, Donna Catalano, Austin Talis, Gyongyi Szabo, and Shashi Bala

Materials and Methods

Histopathological Analysis

Formalin-fixed liver sections were stained with hematoxylin-eosin (H&E) using standard protocols. The slides were analyzed under light microscopy at 100X.

Biochemical Analysis

The liver injury was assessed at the enzymatic level by measuring alanine aminotransferase (ALT) activity from plasma samples using a kinetic method (TECO Diagnostics, CA, USA) as per manufacturer's instructions.

Western Blotting

Extracellular vesicles were lysed in RIPA buffer and checked for CD63 expression by Western blot analysis as described previously[1].

Patient Samples

Human liver samples were obtained from the National Institutes of Health Liver Tissue Cell Distribution System (Minneapolis, MN). Liver tissues were from control subjects, and HCV patients with cirrhosis (n=8-10).

Cell Culture

Hepa1.6 mouse hepatocyte cell line was purchased from ATCC and maintained in a low-glucose DMEM (Thermo Fisher Scientific, MA, USA) containing 10% FBS (HyClone Laboratories, UT, USA) at 37°C in a 5% CO_2 atmosphere as described previously[2, 3]. LX2 cells were cultured in low glucose DMEM medium as described previously[4] and treated with 5ng/ml TGFβ for indicated

times. Cells were washed with 1XPBS twice and cells were lysed in Qiazole (Qiagen, USA) and processed for total RNA extraction (Zymo Research, USA).

miR target analysis and correlation studies

Experimentally validated targets of miR-132 in the liver were predicted and visualized by miRNet and correlation studies were performed using TCGA set.

Transfection

For overexpression of miR-132, cells (macrophages and hepatocytes) were seeded onto 24-well plates and next day, cells were treated either with a negative control mimic #1 or miR-132 mimic (150 pmol) and for inhibition of miR-132, cells were treated either with negative control inhibitor #1 or miR-132 inhibitor (150 pmol) for 24h (Applied Biosystems, CA, USA) using lipofectamine RNAi max reagent (Thermo Fisher Scientific, CA, USA) as described previously[2, 3]. Some cells were either treated or not with 0.1% CCl_4 for the last 6h of experiment as described previously[5]. Cells were washed with 1XPBS for two times and lysed in RNA lysis buffer for total RNA extraction or RIPA buffer for protein extraction and stored at -80°C for further analysis.

Electroporation of miRNA into the exosomes and stimulation of RAW macrophages

Loading of control or miR-132 mimic into the exosomes, isolated from THP1 cells, was performed using our previously optimized protocol[1, 6]. Briefly, exosomes were diluted in Gene Pulser® electroporation buffer (Bio-Rad Laboratories, Berkeley, CA) in 1:1 ratio and miR-132 mimic or negative control mimic (Ambion, Grand Island, NY) at 300 pmol were mixed with exosome suspension containing 1 µg/µl exosomal protein. The suspension was transferred into cold 0.2 cm electroporation cuvettes and electroporated at 150 kV and 100 µF using a Gene pulser II System

(Bio-Rad Laboratories, Berkeley, CA) for electroporation. The exosomes were treated with one unit of RNase H to eliminate free-floating miR and re-isolated using ExoQuick-TC™. These loaded exosomes were co-cultured with RAW macrophages for 12 h. Subsequently, cells were washed to remove the free-floating exosomes and cells were cultured in the fresh DMEM high glucose medium for 24h. As a positive control, some cells were treated with LPS (10ng/ml) for 24h. At the end of stimulation, cells were washed with 1XPBS twice and cells were lysed in Qiazole (Qiagen, USA) and processed for total RNA extraction (Zymo Research, USA).

Statistical Analysis

Based on data distribution, one-way analysis of variance (ANOVA) was used to compare different groups. Mann-Whitney *U* test were performed for comparing two groups. Data are presented as mean ± standard error of mean (SEM). P values less than 0.05 was considered as statistically significant.

Supplementary Table 1: TCGA dataset used for analysis.

Supplementary Table 1.xlsx

Supplementary Table 2: Primer sequences are listed below.

**mMMP12**	Forward	TGGCCATTCCTTGGGGCTGC
	Reverse	GGGGGTTTCACTGGGGCTCCATA
**mFoxo3**	Forward	GGAATCGTACGCCCTCCCG
	Reverse	TGCTCTCTCCTCTCGAGCC
**mSIRT1**	Forward	CGGCTACCGAGGTCCATATAC
	Reverse	CAGCTCAGGTGGAGGAATTGT
**mCOX2**	Forward	GCCTACTACAAGTGTTTCTTTTTGCA
	Reverse	CATTTTGTTTGATTGTTCACACCAT
**mIL1B1**	Forward	TGGACCTTCCAGGATGAGGACA
	Reverse	GTTCATCTCGGAGCCTGTAGTG
**mTGFB1**	Forward	CAAGGGCTACCATGCCAACT
	Reverse	GTACTGTGTGTCCAGGCTCCAA
**mMCP1**	Forward	GCTACAAGAGGATCACCAGCAG
	Reverse	GTCTGGACCCATTCCTTCTTGG
**mVimentin**	Forward	CCCTCACCTGTGAAGTGGAT
	Reverse	TCCAGCAGCTTCCTGTAGGT
**mN-cadherin**	Forward	TGAAACGGCGGGATAAAGAG
	Reverse	GGCTCCACAGTATCTGGTTG
**m18s**	Forward	GCAATTATTCCCCATGAACG
	Reverse	GGCCTCACTAAACCATCCAA

Suplementary Figure 1

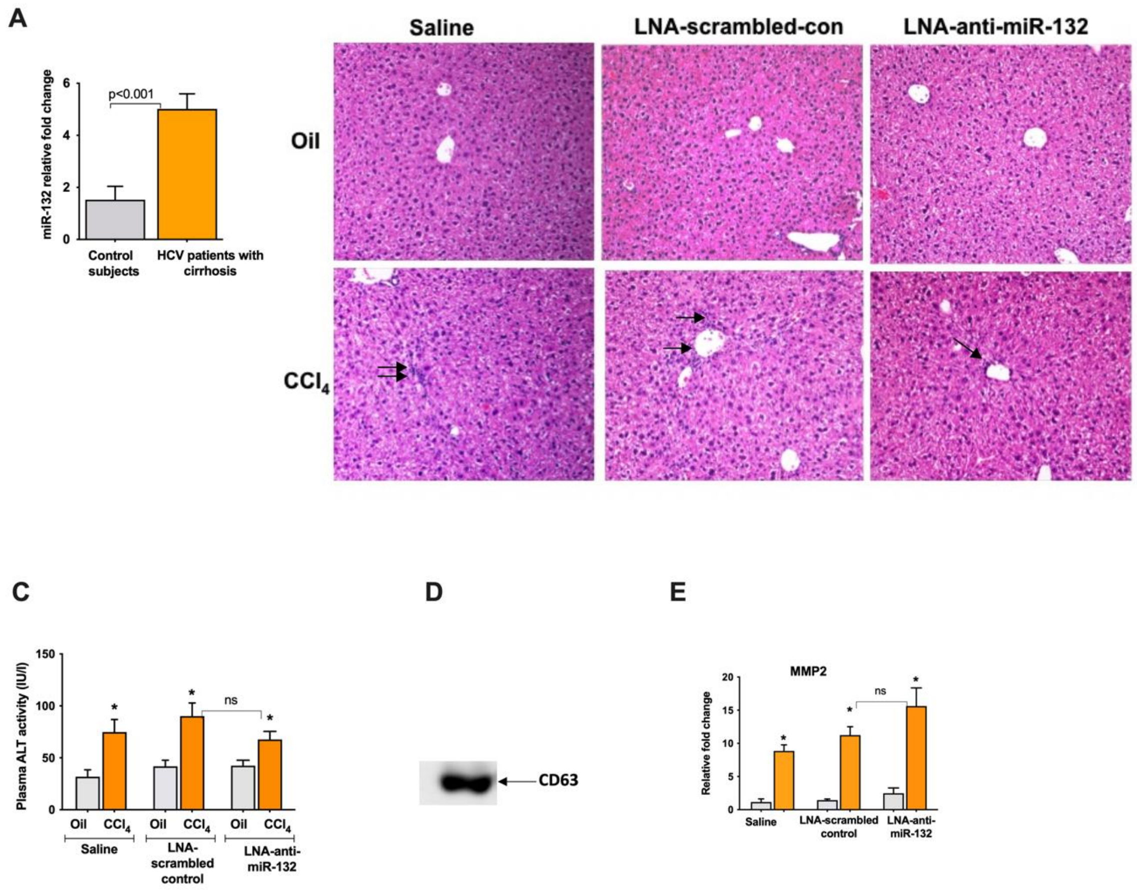

Figure 1. Induction of miR-132 in liver fibrosis/cirrhosis. A) Liver tissues (10mg) of HCV patients with cirrhosis and respective control individuals (n=8-10/group) were used for total RNA extraction using miRNeasy kit. The levels of miR-132 was quantified using TaqMan miR real time PCR assay and RNU48 was used as an internal control. **B)** C57BL/6 male mice (n=8) were injected either with LNA-scrambled control or LNA-anti-miR-132 (15mg/kg) as described in methods. Some mice received either corn oil or CCl_4 (i.p.; 0.6ml/kg of body weight) for indicated times. Representative images of H&E staining of paraffin embedded liver sections. Arrows indicates mononuclear cells. **C)** ALT levels from plasma samples. **D)** CD63 expression from isolated EVs. **E)** MMP2 mRNA transcripts were detected from RNA by real time qPCR and 18S was used to normalize Ct values. Data represent mean ± SEM. Mann-Whitney test or one-way ANOVA was

employed for statistical analysis. * indicates p<0.05 compared to respective control mice. Ns: non significant.

Suplementary Figure 2

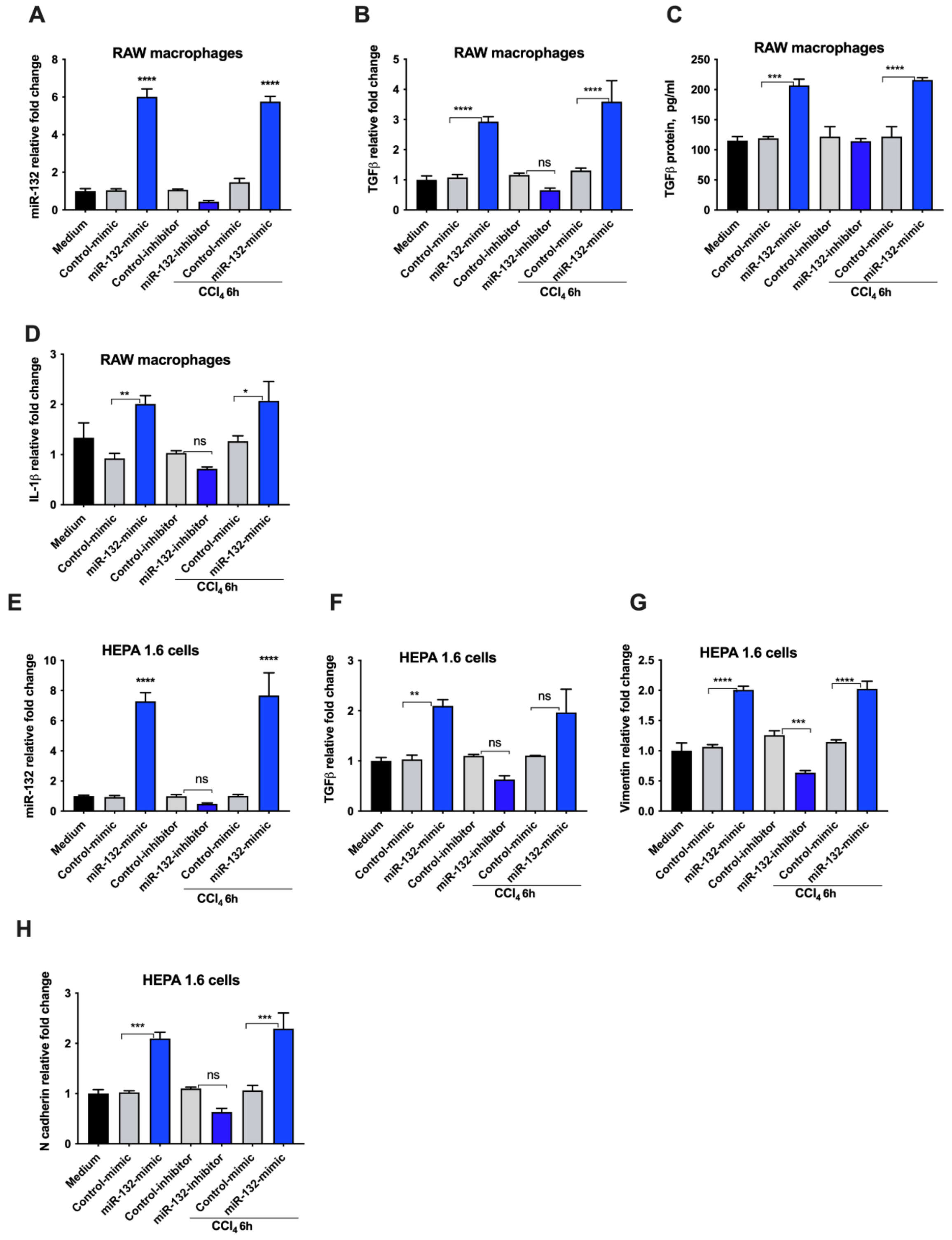

Figure 2. miR-132 regulates the expression of pro-inflammatory and pro-fibrogenic genes. RAW macrophages or Hepa 1.6 hepatocytes were transfected with either control or miR-132 mimic or inhibitor as described in the methods. For the last 6h of transfection, cells were treated or not with 1% CCl_4 and expression of miR-132 (A, E), TGFβ mRNA (B, F) and protein (C) IL-1β mRNA (D) , vimentin (G) and n cadherin (H) was analyzed by real time qPCR and ELISA. Data is shown as mean ± SEM (n=3). Mann-Whitney test or one-way ANOVA was employed for statistical analysis. *, **, ***, **** indicates p<0.05, p<0.005, p<0.0005, p<0.0001. ns non-significant.

Suplementary Figure 3

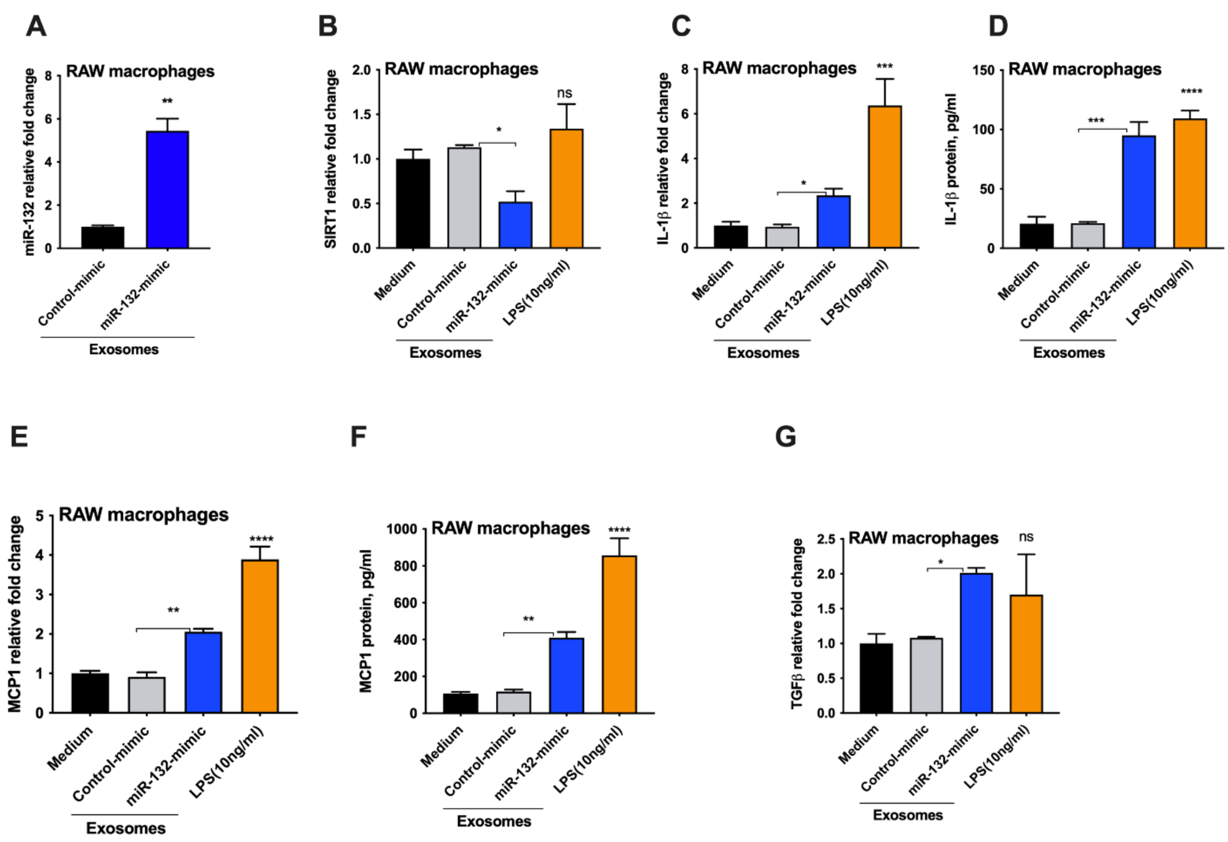

Figure 3. Exosome mediated delivery of miR-132 mimic regulates SIRT1 and inflammatory genes expression in macrophages. Control or miR-132 mimic were loaded into exosomes as

described in the methods. Exosomes were added to naïve RAW macrophages for 12 h and afterwards exosomes were washed off and media was replaced and cultured for 24h. Some cells were treated with10ng/ml LPS for 24h. **A)** miR-132 levels were quantified by real time qPCR and SNORNA-202 was used to normalize Ct values. **B)** SIRT1 **C-D)** IL-1β mRNA and protein levels, **E-F)** MCP1 mRNA and protein levels, and **G)** TGFβ expression was evaluated by real time qPCR and ELISA respectively. 18S was used to normalize Ct values. Data represent mean ± SEM (n=3). one-way ANOVA was employed for statistical analysis. *, **, ***, **** indicates $p<0.05$, $p<0.005$, $p<0.0005$, $p<0.0001$ respectively compared to control cells. Ns: non-significant.

Suplementary Figure 4

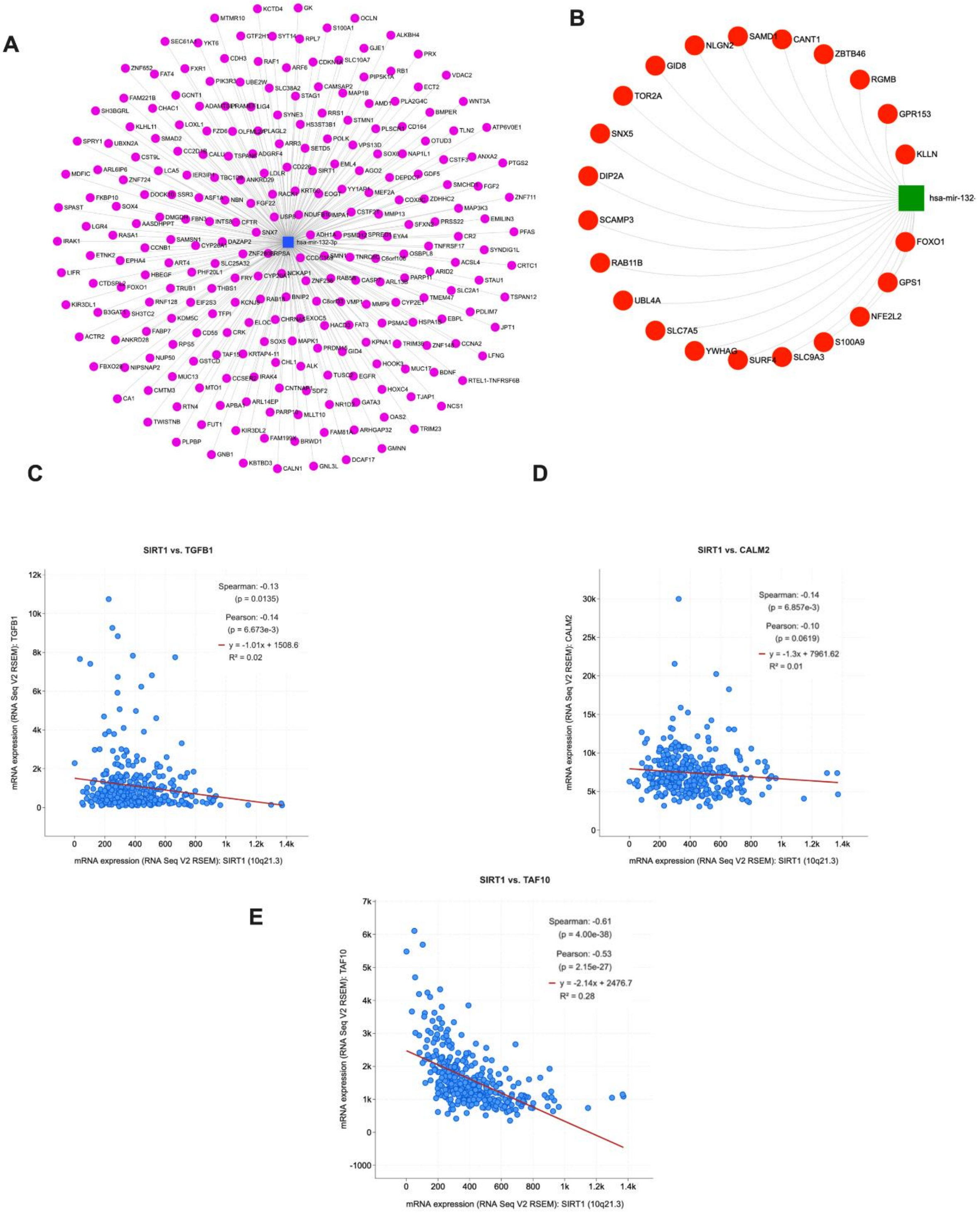

Figure 4. miR-132 target analysis. A) Targets of miR-132 were identified based on experimentally validated miR/mRNA interaction. **B)** Direct hepatocellular carcinoma-related

targets of miR-132 were identified based on experimentally validated miR/mRNA interaction. **C-E)** Correlation between miR-132 target gene SIRT1 was evaluated with fibrogenic markers, TGFβ (C) CALM2 (D) and TAF16 (E).

Supplementary Figure 5

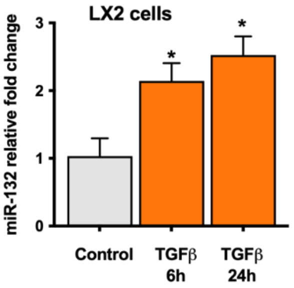

Figure 5. miR expression in hepatic stellate cells. LX2, a human HSC cells were treated with 5ng/ml TGFβ for indicated times and miR-132 levels were quantified and RNU48 was used as an internal control. Data represent mean ± SEM. Mann-Whitney test was employed for statistical analysis. * indicates $p<0.05$ compared to control.

References

1. Momen-Heravi F, Bala S, Kodys K, Szabo G. Exosomes derived from alcohol-treated hepatocytes horizontally transfer liver specific miRNA-122 and sensitize monocytes to LPS. Sci Rep. 2015;5:9991. Epub 2015/05/15. doi: 10.1038/srep09991. PubMed PMID: 25973575; PubMed Central PMCID: PMCPMC4650752.
2. Zhang L, Huang D, Wang Q, Shen D, Wang Y, Chen B, Zhang J, Gai L. MiR-132 inhibits expression of SIRT1 and induces pro-inflammatory processes of vascular endothelial inflammation through blockade of the SREBP-1c metabolic pathway. Cardiovasc Drugs Ther. 2014;28(4):303-11. Epub 2014/06/14. doi: 10.1007/s10557-014-6533-x. PubMed PMID: 24924687.
3. Chen IC, Chiang WF, Huang HH, Chen PF, Shen YY, Chiang HC. Role of SIRT1 in regulation of epithelial-to-mesenchymal transition in oral squamous cell carcinoma metastasis. Mol Cancer. 2014;13:254. Epub 2014/11/27. doi: 10.1186/1476-4598-13-254. PubMed PMID: 25424420; PubMed Central PMCID: PMCPMC4258025.

4. Friedman SL. Hepatic stellate cells: protean, multifunctional, and enigmatic cells of the liver. Physiol Rev. 2008;88(1):125-72. Epub 2008/01/16. doi: 10.1152/physrev.00013.2007. PubMed PMID: 18195085; PubMed Central PMCID: PMCPMC2888531.
5. Bala S, Calenda CD, Catalano D, Babuta M, Kodys K, Nasser IA, Vidal B, Szabo G. Deficiency of miR-208a Exacerbates CCl4-Induced Acute Liver Injury in Mice by Activating Cell Death Pathways. Hepatol Commun. 2020;4(10):1487-501. Epub 2020/10/08. doi: 10.1002/hep4.1540. PubMed PMID: 33024918; PubMed Central PMCID: PMCPMC7527689.
6. Momen-Heravi F, Bala S, Bukong T, Szabo G. Exosome-mediated delivery of functionally active miRNA-155 inhibitor to macrophages. Nanomedicine. 2014;10(7):1517-27. Epub 2014/04/02. doi: 10.1016/j.nano.2014.03.014. PubMed PMID: 24685946; PubMed Central PMCID: PMCPMC4180003.
