# Supplementary material for: Protective effect of LNA-anti-miR-132 therapy on liver fibrosis in mice
Source: Mol Ther Nucleic Acids. 2021 May 14;25:155–67. doi: 10.1016/j.omtn.2021.05.007 (PMC8368790; doi:10.1016/j.omtn.2021.05.007)
Supplement: Document S2. Article plus supplemental information [file mmc3.pdf]

Molecular Therapy
Nucleic Acids
Original Article

Protective effect of LNA-anti-miR-132 therapy on liver fibrosis in mice

Fatemeh Momen-Heravi,[1,2,3] Donna Catalano,[4] Austin Talis,[1,2] Gyongyi Szabo,[5] and Shashi Bala[4,6]

[1]Cancer Biology and Immunology Laboratory, College of Dental Medicine, Columbia University Irving Medical Center, New York, NY, USA; [2]Division of Periodontics, Section of Oral, Diagnostic, and Rehabilitation Sciences, Columbia University College of Dental Medicine, New York, NY, USA; [3]Herbert Irving Comprehensive Cancer Center, Columbia University Irving Medical Center, New York, NY, USA; [4]Department of Medicine, University of Massachusetts Medical School, Worcester, MA 01605, USA; [5]Department of Medicine, Beth Israel Deaconess Medical Center and Harvard Medical School, Boston, MA, USA; [6]KASA BIO, 10405 Old Alabama Road Connector, Suite 201, Alpharetta, GA 30022, USA

microRNAs (miRs) are small regulatory RNAs that are frequently deregulated in liver disease. Liver fibrosis is characterized by excessive scarring caused by chronic inflammatory processes. In this study, we determined the functional role of miR-132 using a locked nucleic acid (LNA)-anti-miR approach in liver fibrosis. A significant induction in miR-132 levels was found in mice treated with CCl_4 and in patients with fibrosis/cirrhosis. Inhibition of miR-132 in mice with LNA-anti-miR-132 caused decreases in CCl_4-induced fibrogenesis and inflammatory phenotype. An attenuation in collagen fibers, α SMA, MCP1, IL-1β, and Cox2 was found in LNA-anti-miR-132-treated mice. CCl_4 treatment increased caspase 3 activity and extracellular vesicles (EVs) in control but not in anti-miR-132-treated mice. Inhibition of miR-132 was associated with augmentation of MMP12 in the liver and Kupffer cells. *In vivo* and *in vitro* studies suggest miR-132 targets SIRT1 and inflammatory genes. Using tumor cancer genome atlas data, an increase in miR-132 was found in hepatocellular carcinoma (HCC). Increased miR-132 levels were associated with fibrogenic genes, higher tumor grade and stage, and unfavorable survival in HCC patients. Therapeutic inhibition of miR-132 might be a new approach to alleviate liver fibrosis, and treatment efficacy can be monitored by observing EV shedding.

INTRODUCTION

Liver homeostasis is crucial for normal liver function, and its disbalance leads to pathogenesis. Long-term alcohol use impairs liver function and results in alcoholic liver disease (ALD). ALD is a multifactorial disease, the spectrum of which ranges from steatosis, hepatitis, fibrosis, and cirrhosis to eventual hepatocellular carcinoma (HCC) if left untreated.[1,2] Studies suggest that ~50% of heavy drinkers eventually develop liver fibrosis/cirrhosis.[3,4] Liver fibrosis is characterized by excessive scarring, caused by chronic inflammatory processes activated during liver diseases of various origins such as alcohol use, non-alcoholic steatohepatitis (NASH), and hepatitis C (HCV) infection.[5–7] Liver fibrosis is an integral part in the progression of chronic liver disease, ultimately leading to cirrhosis and HCC.[8] Impairment in pathways involved in inflammation, tissue repair, and deposition of extracellular matrix can lead to the development of liver fibrosis.[9] Activation of quiescent hepatic stellate cells (HSCs), characterized by HSC morphological transition to myofibroblast-like cells, is the central event in the pathogenesis of hepatic fibrosis.[10] Moreover, epithelial-mesenchymal transition (EMT) in HSCs and liver parenchymal epithelial cells also contributes to fibroblast transition.[9]

microRNAs (miRs), a class of small non-coding RNAs, have emerged as new crucial regulators of cellular processes.[11] miR-132 is extensively studied in the brain, and its dysregulation is found in several neurological disorders.[12,13] Emerging studies, including ours, suggest a role of miR-132 in other organs.[13] In our previous studies, we showed induction of miR-132 in a mouse model of ALD both in isolated Kupffer cells (KCs) and hepatocytes after chronic alcohol feeding.[14] Recent studies indicate a role of miR-132 in the pathogenesis of gastrointestinal cancers.[15] Several miRs are shown to be involved in liver fibrosis,[16–18] though, to date, the functional roles of only a few miRs were revealed in liver disease. Therefore, studying the loss and gain of function for other miRs is necessary for further mechanistic insight into miR-dependent regulation of liver function and homeostasis. miR-132 and miR-212, for instance, are derived from the same non-coding gene, share the same seed sequence, and are similarly highly conserved among vertebrates;[13] however miR-132 is unique in its regulation of numerous chromatin-remodeling factors involved in acetylation, deacetylation, and oxidative stress.[19,20]

As chronic alcohol abuse leads to liver fibrosis/cirrhosis, we postulate that miR-132 has a causative role in this process. Consistent with our hypothesis, we found a ~4-fold induction of miR-132 in the livers of alcoholic patients with fibrosis/cirrhosis. A significant induction of miR-132 was found in the livers of mice treated with CCl_4, a long-established inducer of liver fibrosis.[21] CCl_4-induced liver fibrosis was found to be attenuated in mice given LNA (locked nucleic acid)-anti-miR-132. Attenuation in pro-fibrotic gene expression and caspase activation were observed in mice receiving LNA-anti-miR-132.

Received 24 September 2020; accepted 7 May 2021;
https://doi.org/10.1016/j.omtn.2021.05.007.

Correspondence: Shashi Bala, PhD, KASA BIO, 3941 Holcomb Bridge Road, Suite 301, Peachtree Corners, GA 30092, USA.
E-mail: shashib@kasa-bio.com

This is an open access article under the CC BY license (http://creativecommons.org/licenses/by/4.0/).

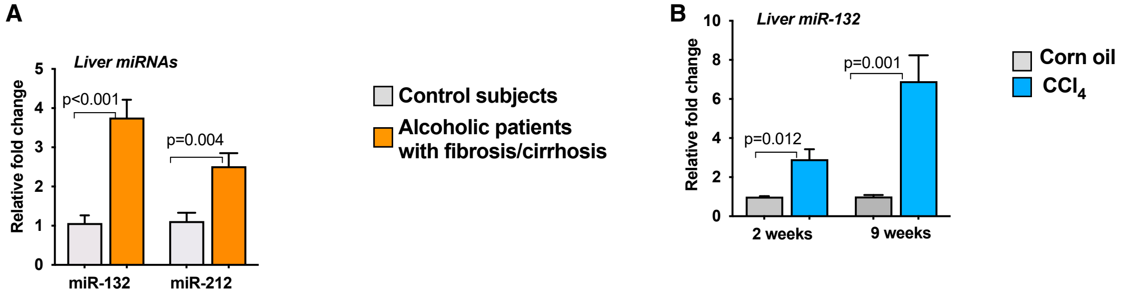

Figure 1. Induction of miR-132 in liver fibrosis

(A) Liver tissues (10 mg) of control individuals and patients with either alcoholic fibrosis or cirrhosis (n = 8/group) were used for total RNA extraction using miRNeasy kit. The levels of miR-132 and miR-212 were detected using TaqMan miR real-time PCR assay, and RNU48 was used as an internal control. (B) C57BL/6 male mice (n = 8) received either corn oil or CCl_4 for indicated times. Total RNA from the liver was used for miR-132 analysis as described in Materials and methods, and SnoRNA-202 was used as internal control. Data represent mean ± SEM. Mann-Whitney test was employed for statistical analysis.

At the cellular level, miR-132 was increased in both hepatocytes and KCs isolated after CCl_4 treatment. We also found higher levels of miR-132 in HCC samples compared to controls. High miR-132 levels were associated with higher tumor grade and stage and significantly unfavorable survival in HCC patients. Our results suggest that anti-miR-132-treated mice are protected from CCl_4-induced liver fibrosis, suggesting a role for miR-132 in fibrogenic processes.

RESULTS

Induction of miR-132 in the fibrotic livers of alcoholic patients and in CCl_4-induced liver fibrosis mouse model

Previously, we showed induction of miR-132 in the liver, isolated KCs, and hepatocytes after chronic alcohol feeding in mice.[14] Chronic liver injury caused by dietary intake (alcohol or NASH) or viral infections can lead to downstream fibrosis and cirrhosis;[22] therefore, we checked the levels of miR-132 in the livers of alcoholic patients with fibrosis/cirrhosis. A significant induction in miR-132 expression was found in the livers of alcoholic patients with fibrosis/cirrhosis, as seen in Figure 1A. Since miR-132 and miR-212 are closely correlated and encoded in the same intron of a small non-coding gene, we examined miR-212 levels in these samples. Although both miR-132 and miR-212 were significantly increased in patients with alcoholic cirrhosis ($p < 0.05$), consistent with previous reports[13] we found a higher induction of miR-132 compared to miR-212 (~2.5 fold) (Figure 1A). Additionally, a significant increase in miR-132 levels was found in the cirrhotic livers of HCV patients (Figure S1A). Therefore, we focused our mechanistic studies on miR-132. Next, we checked the levels of miR-132 in a CCl_4-induced liver fibrosis mouse model. We found a significant and sustained induction of miR-132 in the livers of mice after administration of CCl_4 for both 2 and 9 weeks (Figure 1B).

Inhibition of miR-132 with LNA-anti-miR-132 attenuates CCl_4-induced liver fibrosis

To determine the role of miR-132 in liver fibrosis, we used a mouse model of CCl_4-induced liver fibrosis. Currently, there is no widely accepted mouse model of ALD that represents the liver fibrosis/cirrhosis observed in alcoholic patients. The CCl_4-induced liver fibrosis mouse model is widely used and has proven to be robust and reproducible, able to mimic the histological, biochemical, and molecular changes associated with the development of fibrosis.[23] We inhibited miR-132 function in mice using miRCURY LNA inhibitor (LNA-anti-miR-132). The schematic of LNA-anti-miR-132 treatment schedule and CCl_4 administration is shown in Figure 2A.

CCl_4 treatment increased the hepatic miR-132 levels in saline- or scrambled-control-treated mice, and miR-132 was almost undetected in the treatment group (LNA-anti-miR-132) across both oil- and CCl_4-treated mice (Figure 2B). CCl_4-induced increase in miR-212 was prevented in mice treated with miR-132 inhibitor (Figure 2C). Consistently, this effect was specific to LNA-anti-miR-132 and was not observed in LNA-scrambled control. The liver damage assessed by histological evaluation of H&E staining revealed fewer mononuclear cells (Figure S1B) and decreased ALT levels (Figure S1C) in the livers of LNA-anti-miR-132-treated mice after CCl_4 treatment. Evaluation of Sirius Red-stained slides showed that administration of LNA-anti-miR-132 attenuated the fibrosis phenotype of liver tissue in CCl_4 liver injury, as characterized by less fibrous tissue compared to the controls (Figure 2D). CCl_4 treatment was found to have induced the expression of fibrogenic gene transcripts (collagen 1α, TIMP1, and TGFβ) in LNA-scrambled control-treated mice, whereas LNA-anti-miR-132 treatment attenuated CCl_4-induced increase in collagen 1α and inhibited the induction of TIMP1 and TGFβ transcripts (Figure 2E). These findings were further corroborated via decreased α smooth muscle actin protein levels in the livers of LNA-anti-miR-132-treated mice compared with the LNA-scrambled control-treated mice in CCl_4-induced liver injury (Figure 2F).

LNA-anti-miR-132 attenuates CCl_4-induced release of extracellular vesicles and caspase 3 activity

It has been well established that upon induction of cell stress, cells produce EVs,[24,25] and here, consistent with those findings, we observed increases in the total number of circulating EVs produced, which ranged from exosomes (<200 nm) to larger microvesicles (>200 nm) (Figure 3A). The mean diameters of EVs were 320 nm for CCl_4-treated mice and 117 nm for oil-treated mice (Figure 3A).

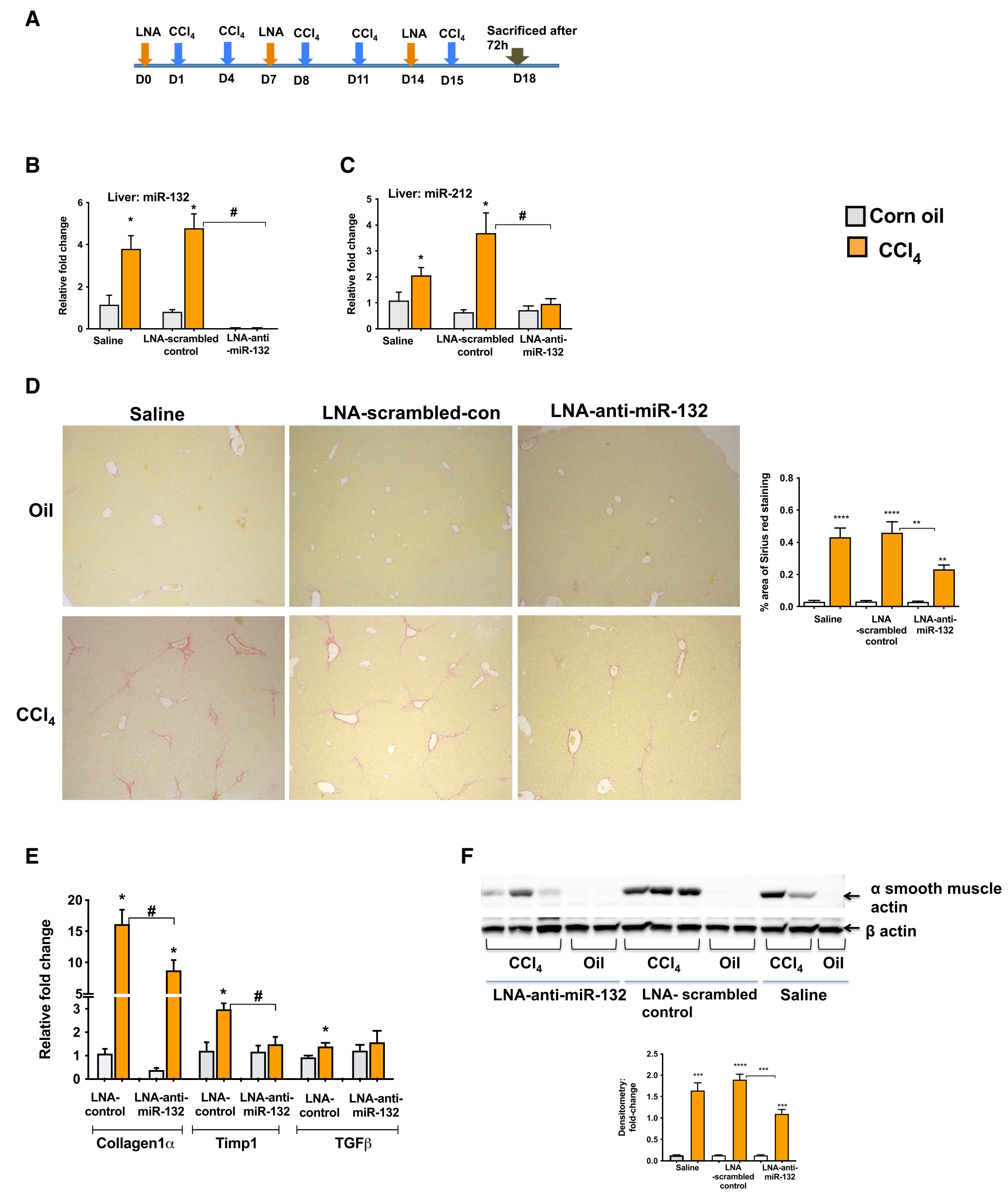

Figure 2. LNA-anti-miR-132 delivery in mice

(A) C57BL/6 male mice (n = 8) were injected either with LNA-scrambled control or LNA-anti-miR-132 (15 mg/kg) or saline intraperitoneally (i.p.) as shown. Some mice received either corn oil or CCl_4 (i.p.; 0.6 mL/kg of body weight) for indicated times. (B and C) RNA isolated from the liver was used to determine miR-132 and miR-212 expression by quantitative real-time PCR using TaqMan microRNA assay. SnoRNA-202 was used as internal control. (D) Sirius Red staining of paraffin-embedded liver sections. Left panel depicts representative slides observed under light microscopy (100×), and the right panel shows ImageJ quantification of the stained area. (E) RNA isolated from the liver was used to determine the expression levels of collagen1α, TIMP1, and TGFβ. (F) 20 μg of whole liver lysate protein was used to determine α smooth muscle actin expression by western blot (left panel). β actin was used as a loading control. Densitometry units are shown as fold change compared to oil-saline-treated mice after normalization (lower panel). Data represent mean ± SEM. Mann-Whitney test or one-way ANOVA was employed for statistical analysis. *$p < 0.05$ compared to oil-scrambled control-treated mice. **$p < 0.005$, ***$p < 0.0005$, ****$p < 0.0001$. #$p < 0.05$ compared to LNA-scrambled control-treated mice after CCl_4 treatment.

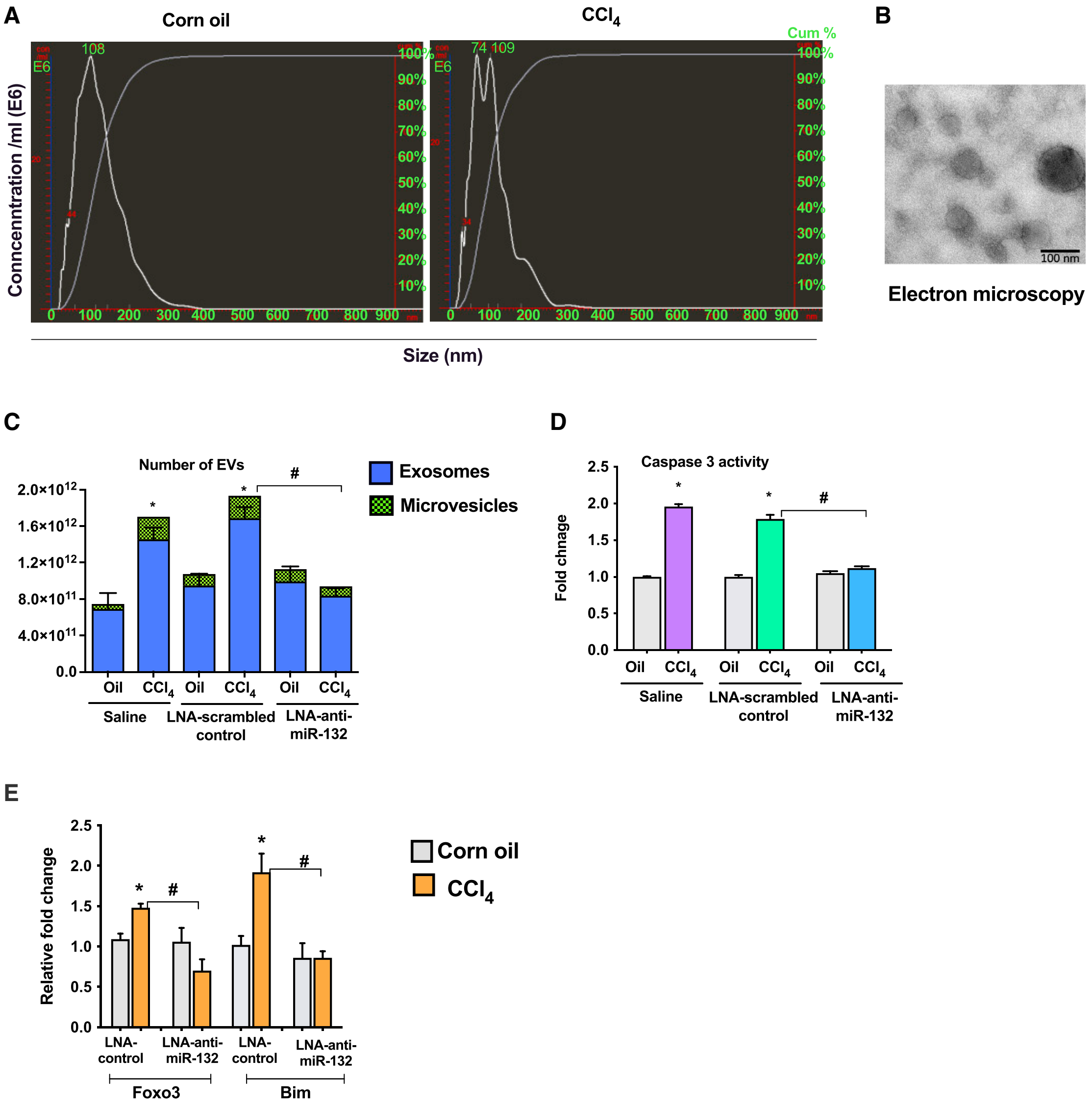

Figure 3. miR-132 inhibition prevents CCl_4-induced increase in EVs in the plasma

Mice received the treatments as described in Figure 2. Total number of extracellular vesicles (EVs) was measured from plasma using NanoSight as described in the Materials and methods (n = 5). (A) Size distribution of vesicles isolated from plasma after CCl_4 treatment by NTA analysis. (B) Extracellular vesicles were isolated using ExoQuick solution as described in the Materials and methods and used for electron microscopy. Representative picture is shown. (C) NTA analysis of extracellular vesicles after miR-132 inhibition. (D) Caspase 3 activity was determined from liver cell lysate using colorimetric assay as described in the Materials and methods . Fold change was calculated using saline-oil-treated mice. (E) Expression of Foxo3 and Bim was quantified by real-time PCR, and 18S was used to normalize Ct values. Data are shown as mean ± SEM. Mann-Whitney test or one-way ANOVA was employed for statistical analysis. *$p < 0.05$ compared to mice treated with oil and saline. #$p < 0.05$ compared to LNA-scrambled control-treated mice after CCl_4 treatment.

The isolated EVs were visualized with electron microscopy and showed the morphology of extracellular vesicles described before[26] (Figure 3B) as well as exhibiting CD63 expression (Figure S1D). Administration of LNA-anti-miR-132 significantly reduced production of EVs in CCl_4-treated mice compared to LNA-scrambled control as well as restored the production of exosomes to the level of baseline oil-treated mice (Figure 3C). No change in the size of EVs was observed in the plasma of LNA-anti-miR-132-treated mice. Caspase 3 is shown to play an important role in the promotion of fibrosis and associated cell damage as well as the production of apoptotic EVs.[27,28] Thus, we hypothesized that LNA-anti-miR-132 can exert a protective effect on caspase 3 activity. Interestingly, CCl_4-induced caspase 3 activity was significantly reduced after administration of LNA-anti-miR-132 but not LNA-scrambled control (Figure 3D). The pro-apoptotic genes Foxo3 and Bim were induced in LNA-scrambled control CCl_4-treated mice, whereas increases in these

genes were inhibited after administration of LNA-anti-miR-132 (Figure 3E).

LNA-anti-miR-132 suppresses the expression of pro-inflammatory and pro-fibrogenic genes

The inflammatory responses play an important role in fibrogenesis, as persistent inflammation primes fibrotic events.[6] An increase in CD68, a macrophage inflammatory marker, and MCP1 chemokine was found in LNA-scrambled control mice, and LNA-anti-miR-132 treatment prevented an induction in these genes after CCl_4 treatment (Figure 4A). Further, expression of inflammatory mediators IL-1β and Cox2 was induced in LNA-scrambled CCl_4-treated mice, and an attenuation in these genes was observed after LNA-anti-miR-132 treatment (Figure 4A). Matrix metalloproteases (MMPs) are shown to play a key role in repairing connective tissue damage. Macrophage metalloelastase-12 (MMP-12) plays an active role in the turnover of elastin,[29,30] and we found substantially increased levels of MMP-12 transcripts in the liver after CCl_4 treatment (Figure 4B). Furthermore, mice treated with LNA-anti-miR-132 showed a significantly higher induction of MMP-12 compared to mice treated with LNA-scrambled control after CCl_4 treatment ($p < 0.05$) (Figure 4B). Consistently, the active forms of MMP-12 were increased to a greater extent in the livers of mice treated with LNA-anti-miR-132 as compared to mice treated with LNA-scrambled control after CCl_4 treatment (Figure 4C). We also found enhanced levels of MMP2 transcripts in LNA-anti-miR-132-CCl_4-treated mice (Figure S1E). Further, protein levels of vimentin, an inducer of EMT process, were attenuated in LNA-anti-miR-132-treated mice compared to LNA-scrambled controls after CCl_4 treatment (Figure 4D). SIRT1, a validated miR-132 target, is involved in inflammatory and fibrotic pathways, and decreased expression of SIRT1 was reported in liver fibrosis.[31] Our results indicated a decrease in SIRT1 expression after CCl_4 treatment in LNA-scrambled mice, and LNA-anti-miR-132 treatment rescued the reduction in SIRT1 (Figure 4E).

Induction of miR-132 expression in KCs and hepatocytes after CCl_4 treatment

To determine the cellular source of the hepatic miR-132 upregulation, we isolated KCs and hepatocytes after 2 weeks of CCl_4 treatment. Our results indicated an increase of miR-132 (Figure 5A) and miR-212 (Figure 5B) in isolated KCs after CCl_4 administration. LNA-anti-miR-132 treatment significantly reduced miR-132 levels in KCs (Figure 5A), whereas miR-212 expression was found to have decreased to that of oil-treated mice (Figure 5B). miR-132 expression was significantly increased in hepatocytes after CCl_4 challenge, and administration of LNA-anti-miR-132 decreased levels of miR-132 more than 20-fold (Figure 5C). CCl_4-induced increase in miR-212 was prevented in hepatocytes isolated from LNA-anti-miR-132-treated mice, suggesting a master regulatory role of miR-132 in liver fibrogenesis (Figure 5D).

Upon discovering augmentation of MMP-12 in the livers of mice treated with LNA-anti-miR-132, we evaluated MMP-12 expression and found an increase in MMP-12 in isolated KCs after CCl_4 treatment (Figure 5E). CCl_4-induced increase in MMP-12 was further amplified in KCs isolated from LNA-anti-miR-132-treated mice (Figure 5E).

To determine the mechanistic role of miR-132, we performed *in vitro* studies using RAW 264.7 mouse macrophages. An induction in miR-132 expression was observed in cells treated with 0.01% CCl_4, and, regardless of CCl_4 treatment, miR-132 levels were induced in the presence of miR-132 mimic and reduced in the presence of miR-132 inhibitor (Figure 5F). Similarly, regardless of CCl_4 treatment, SIRT1 levels were shown to vary indirectly with miR-132 expression (Figure 5G). miR-132 overexpression increased IL-1β mRNA (Figure 5H) and protein levels (Figure 5I) compared to negative control mimic-treated cells with or without CCl_4 treatment. A decrease in IL-1β mRNA (Figure 5H) and protein levels (Figure 5I) was found in cells treated with miR-132 inhibitor compared to negative control inhibitor-treated cells after CCl_4 treatment. Similar findings were observed for TGFβ, wherein miR-132 mimic induced TGFβ levels (Figures S2A–S2D). Further, in Hepa1.6 mouse hepatocytes, miR-132 overexpression resulted in increases in TGFβ, vimentin, and N-cadherin expression (Figures S2E–S2H). No cellular toxicity after CCl_4 treatment was found. The findings of miR-132 regulating SIRT1 were further confirmed by simulation experiments, wherein miR-132 mimic-loaded exosomes were cultured with naive RAW macrophages (Figures S3A–S3G). A reduction in SIRT1 expression was found in cells treated with miR-132 mimic-loaded exosomes compared to control mimic-loaded exosomes (Figure S3B). Conversely, an induction in IL-1β (mRNA and protein), MCP1 (mRNA and protein), and TGFβ mRNA was found in cells treated with miR-132 mimic-loaded exosomes (Figures S3C–S3G).

miR-132 expression is induced in HCC and correlates with fibrogenic and oncogenic genes

As liver fibrosis is one of the risk factors for HCC,[32] we next assessed the expression of miR-132 in HCC in the cancer genome atlas (TCGA) data. We found higher expression of miR-132 in HCC tumor tissue compared to the controls ($p < 0.05$) (Figure 6A). miR target analysis was performed to identify mRNAs that are targeted by miR-132, and our analysis showed implication of important mediators of fibrogenesis, carcinogenesis, and HCC pathogenesis (Figures S4A and S4B). An association between miR-132 and fibrogenic genes was performed in the TCGA data, and the analysis revelated miR-132 expression was inversely correlated with SIRT1 levels (Figure 6B) and positively correlated with TGFβ (Figure 6C), TMIP1 (Figure 6D), COL41α (Figure 6E), AFP (Figure 6F), and LAMB1 (Figure 6G) expression. TCGA analysis also revealed an inverse correlation between SIRT1 and several of fibrotic genes, such as TGFβ, CALM2, and TAF10 (Figure S3E).

Further, higher expression of miR-132 was associated with *TP53* mutation in HCC (Figure 6H) and was also associated with higher tumor grade and stage (Figures 6I and 6J). High expression of miR-132 in HCC patients was associated with significantly lower survival

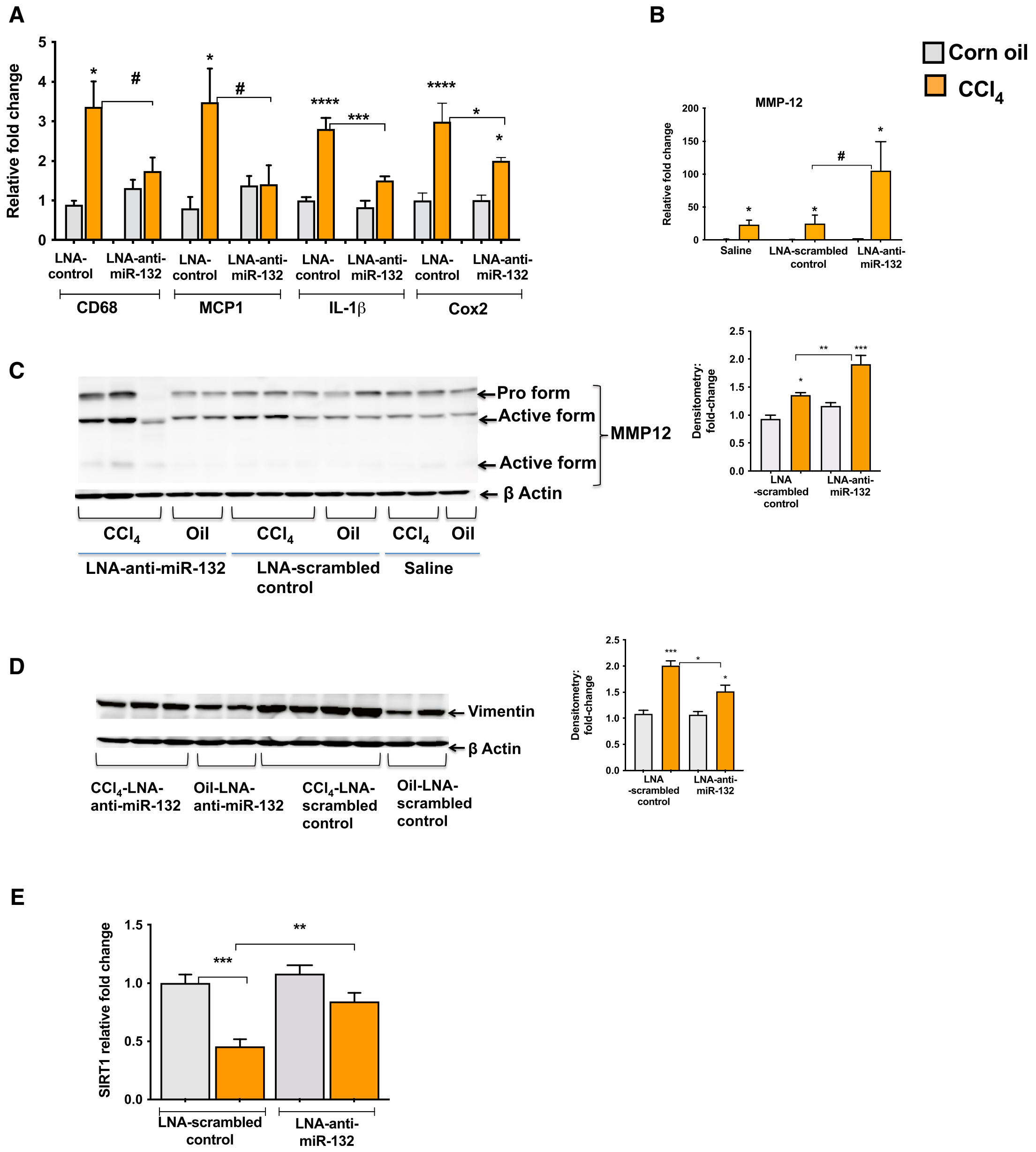

Figure 4. miR-132 inhibition attenuates CCl_4-induced increase in inflammatory and fibrogenic gene expression

Mice received the treatments as described in Figure 2. (A and B) Total RNA was extracted and used for quantification of CD68, MCP1, IL-1β, Cox2, and MMP-12 by real-time PCR, and 18S was used to normalize Ct values. (C and D) 20 μg of whole liver lysate was used to determine the protein levels of MMP-12 and vimentin, and β actin was used as a loading control. Densitometry units are presented as fold change compared to oil-LNA-scrambled control-treated mice after normalization (right panels). (E) SIRT1 expression was quantified by quantitative real-time PCR. Data represent mean ± SEM. Mann-Whitney test or one-way ANOVA was employed for statistical analysis. $^*p < 0.05$ compared to oil-saline-treated mice. $^{**}p < 0.005$, $^{***}p < 0.0005$, $^{****}p < 0.0001$. #p < 0.05 compared to LNA-scrambled control-treated mice after CCl_4 treatment.

compared to patients with low levels of miR-132 ($p = 0.029$) (Figure 6K).

DISCUSSION

Liver fibrosis is an integral part of the progression of chronic liver disease, is a risk factor for HCC, and causes substantial morbidity and mortality throughout the world.[33] To date, there is no successful therapy for the treatment of liver fibrosis, and the most effective treatment remains removal of the causative agent, which is not always possible. Because each miR can potentially regulate several hundred targets,[34] pharmacological modulation of miRs has the potential to treat complex diseases, including liver fibrosis, that are otherwise difficult to

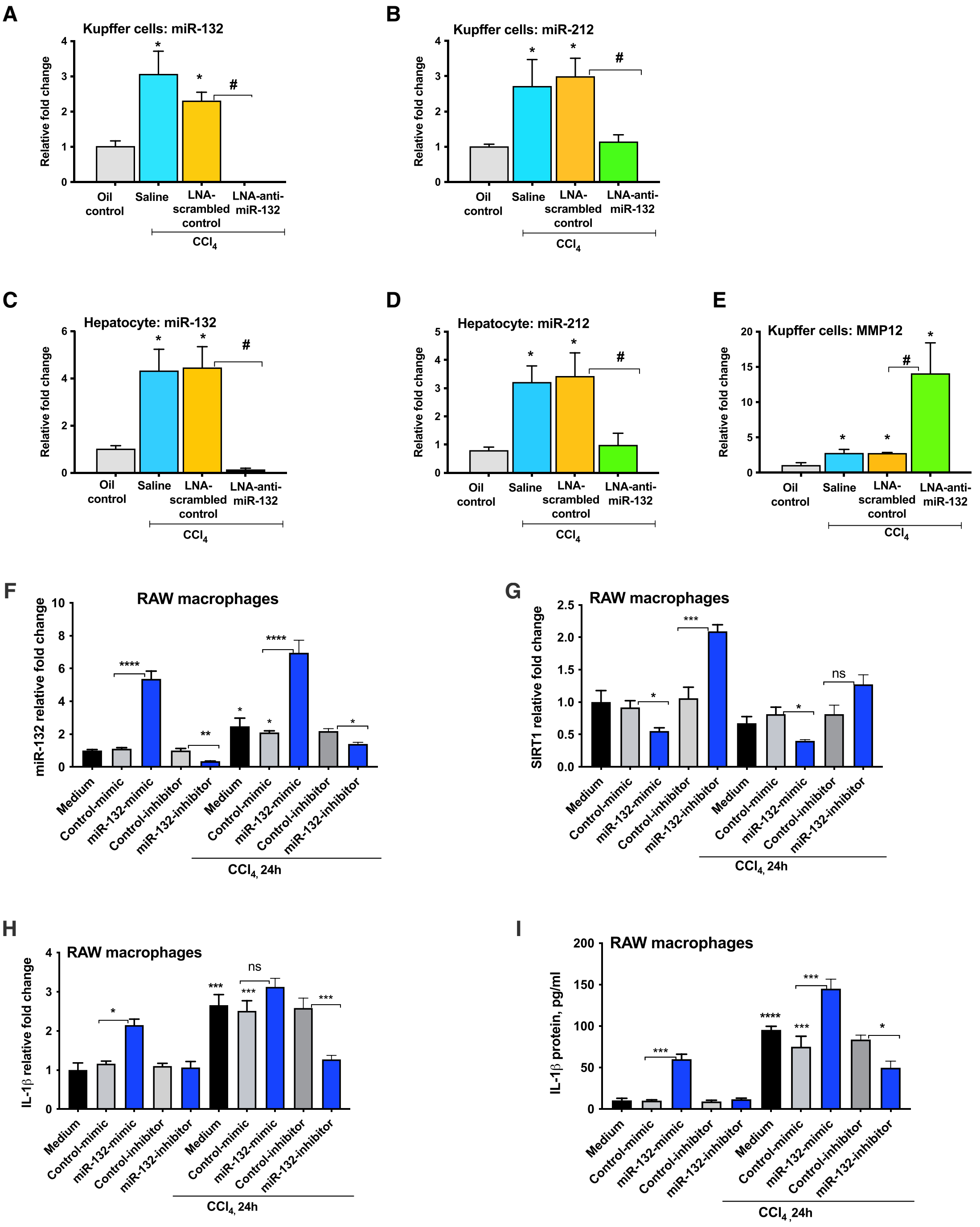

Figure 5. miR-132 increase in Kupffer cells (KCs) and hepatocytes after CCl_4 treatment

Mice received the treatments as described in PFigure 2. (A–D) Total RNA was extracted from KCs and hepatocytes, and expression of miR-132 (A and C) and miR-212 (B and D) was quantified using TaqMan microRNA assay. SnoRNA-202 was used as internal control. (E) MMP-12 expression was measured from KCs using quantitative real-time PCR, and 18S was used to normalize Ct values. Fold change was calculated using cells isolated from saline-oil-treated mice. (F–I) RAW macrophages were transfected with either control or miRNA-132 mimic or inhibitor, as described in the Materials and methods . For the last 24 h of transfection, cells were either treated or not with 0.1% CCl_4, and expression of miR-132 (F), SIRT1 (G), IL-1β mRNA (H), and protein (I) was analyzed by quantitative real-time PCR and ELISA. Data are shown as mean ± SEM (n = 3). Mann-Whitney test or one-way ANOVA was employed for statistical analysis. *$p < 0.05$ compared to cells isolated from saline-oil-treated mice (A–E). **$p < 0.005$, ***$p < 0.0005$, ****$p < 0.0001$ (F–I). ns, non-significant.

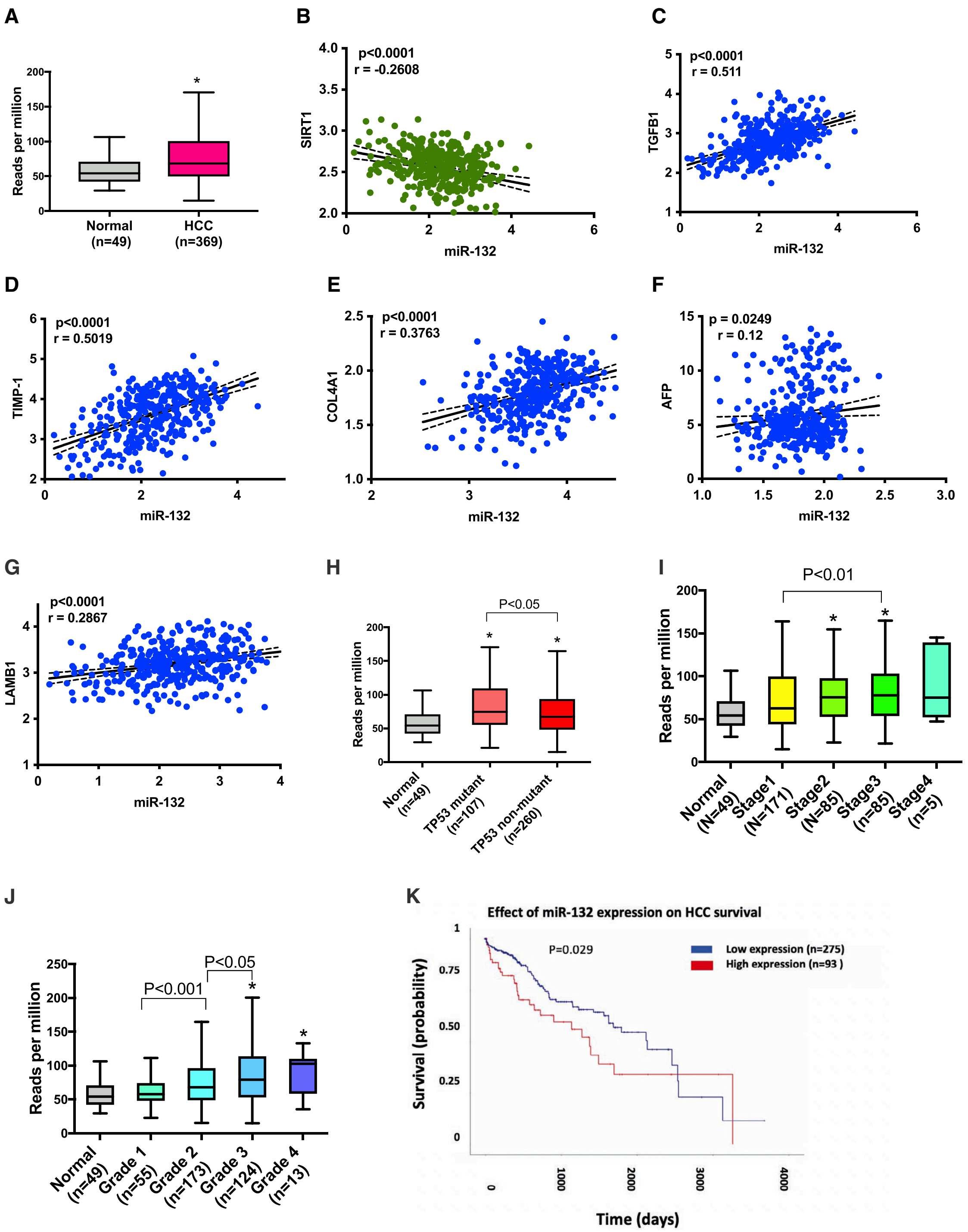

Figure 6. miR-132 is elevated in hepatocellular carcinoma (HCC)

(A) Level of miR-132 in HCC and normal liver tissue in TCGA data (n = 369 HCC; n = 49 normal). (BG) Correlation between miR-132 and fibrogenic genes in TCGA dataset, SIRT1 (B), TGFβ1 (C), TMIP-1 (D), COL4A1 (E), AFL (F), and LAMBI (G). (H) Levels of miR-132 in *TP53* mutant HCC and *TP53* non-mutant HCC (n = 107, *TP53* mutant; and n = 260, *TP53* non-mutant). (I) Levels of miR-132 in different stages of HCC. (J) Levels of miR-132 in different grades of HCC. (K) Kaplan-Meier survival curves of miR-132-high tumors versus miR-132-low tumors. $^{*}p < 0.05$. Correlation between miR-132 and mRNA was quantified with Pearson's correlation coefficient, and correlation coefficients with p value ≤ 0.05 were considered statistically significant. The Mann-Whitney U test was used to compare expression between different groups of samples.

cure using traditional small molecule approaches. Here, employing miR-based therapy, we were able to attenuate the detrimental effects of CCl_4-induced liver fibrosis, a robust model in the study of fibrosis. We found significantly decreased levels of liver fibrosis as characterized by both phenotypic and molecular expression. Importantly, administration of LNA-anti-miR-132 produced a sustainable protective effect against induction of liver fibrosis. LNA-anti-miR-132 provided significant protection against the induction of pro-inflammatory and pro-fibrogenic genes in the CCl_4-treated mice compared to the LNA-scrambled control.

Though studies have suggested a role for miR-132 in fibrosis, to our knowledge this is the first study that mechanistically addresses the role for miR-132 in liver fibrosis. Previous studies have shown that mice treated with an antagomir-132 had attenuation in cardiac fibrosis[35] as well as reduced renal myofibroblast proliferation, resulting in slowed progression of renal fibrosis.[36] miR-132 is known to have a broad regulatory role in the progression of hepatic steatosis, with suppression of multiple miR-132 targets.[37] In this study, we used the CCl_4-induced liver fibrosis mouse model to mimic the molecular and cellular features of liver fibrosis and evaluate potential roles of miR-132 in liver fibrosis and subsequent miR-based treatments. *In vivo* delivery of miRs is challenged by low bioavailability and high sensitivity of RNA interference to enzymatic degradation.[38] LNA includes a class of bicyclic RNA analogs in which the furanose ring in the sugar-phosphate backbone is chemically locked in an RNA mimicking N-type (C3′ endo) conformation by the introduction of a 2′-O,4′-C methylene bridge.[39] These modifications lead to increased nuclease resistance and increased binding affinity of anti-miR oligonucleotides to their target miRs.[40,41] In the present study, using LNA technology allowed us to introduce more robust, stable, and specific inhibition of miR-132 *in vivo*, leading to a significant reversal of fibrosis phenotype and associated molecular changes.

Knowledge of the participation of EVs in pathogenesis of liver disease has expanded greatly in recent years.[42,43] Production of EVs can be induced by many factors, including extracellular stimuli such as microbial attack, cellular injuries, and other stress conditions.[44] Oxidative stress has been linked to increased levels of miR-132 as well as production/release of EVs.[45,46] In this study, we found increased numbers of EVs after induction of liver injury in the mouse model. Administration of LNA-anti-miR-132 attenuated EV production in CCl_4-treated mice, highlighting the efficacy of miR-132 therapy in ameliorating cellular injury and preventing unwanted downstream activity of released EVs. Ethanol-associated activation of caspase 3 and subsequent EV release have been linked to the pro-inflammatory transition of liver macrophages,[47] and here we show that CCl_4-associated inhibition of caspase 3 in LNA-anti-miR-132-treated mice resulted in decreased production of EVs, indicating a mechanistic role of *CASP3* in injury-induced production of EVs. Sirois et al.[28,48] demonstrated a caspase 3-dependent formation of MVBs and the release of apoptotic exosomes in endothelial cells. These data indicate the utility of EVs as potential biomarkers for diagnosis of liver fibrosis as well as monitoring response to therapy.

MMP-12 is highly expressed in macrophages and contributes to elastin degradation.[30] It plays a protective role in liver fibrosis, as MMP-12-deficient mice exhibited more liver fibrosis.[30] We found enhanced expression of MMP-12 in the liver and KCs after LNA-anti-miR-132 treatment and overall protection from liver fibrosis. The regulation of MMPs is complex, and MMP12 is regulated at various levels.[49] miR-132 does not target MMP-12 directly, so it is likely that miR-132 is exerting secondary effects on MMP-12 either via modulating transcription factors or regulation of genes involved in MMP12 regulation. Our TCGA analysis revealed a positive correlation of miR-132 and TIMP1, and we found no increase in TIMP1 transcripts in LNA-anti-miR-132-treated mice, suggesting a role for miR-132 in the fine tuning of fibrotic gene expression.

Our *in vitro* and *in vivo* studies suggest that miR-132 regulates genes involved in fibrogenesis, such as SIRT1, TGFβ, IL-1β, and MCP1. Others have demonstrated a role of miR-132 in promoting inflammation.[50,51] SIRT1, a validated miR-132 target, is shown to be involved in EMT via regulation of mesenchymal markers vimentin and N-cadherin,[52] and we found decreased SIRT1 and vimentin levels after LNA-anti-miR-132 treatment in mice. Further, induction in vimentin and N-cadherin was found in miR-132-overexpressed hepatocytes.

Our results suggest that TGFβ treatment caused induction in miR-132 expression in LX2 cells (human HSC cell line) (Figure S5). Our finding is similar to studies where upregulation of miR-132 was reported in activated human and rat HSCs.[53,54] miR-132 was shown to modulate TGFβ and immune signaling,[34,43] and our *in vitro* studies suggest a regulation of TGFβ, IL-1β, and MCP1 by miR-132. Based on these findings, it is likely that miR-132 concurrently regulates multiple regulatory genes involved in inflammatory and fibrotic changes. The contribution of miR-132 at the cellular level (KCs, hepatocytes, or HSCs) would be an upcoming area of research.

Intriguingly, miR-132 inhibition in CCl_4-treated mice also prevented induction of miR-212. One study has suggested a role for miR-212 in liver fibrosis,[55] though further studies are needed to decipher the contribution of miR-132 and/or miR-212. As liver fibrosis is an established risk factor for HCC, and miR-132 targets many important genes that play roles in carcinogenesis,[56] we found increased levels of miR-132 in the HCC tumor tissue as compared to the control, and high levels of miR-132 were associated with higher-grade and -stage tumors. HCC patients with high tumoral levels of miR-132 had significantly lower survival compared to the patients with low levels of miR-132. These data indicate a possible role for miR-132 in HCC pathogenesis and highlight the utility of miR-132 as an HCC biomarker. Interestingly, miR-132 expression showed correlation with genes related to fibrogenesis. In concordance with our finding of increased miR-132 and decreased SIRT1 in liver fibrosis, an inverse correlation between miR-132 and SIRT1 expression was found in HCC samples in the TCGA database. Others have shown decreased SIRT1 levels in mouse models of liver fibrosis, in patients with cirrhosis, and in activated HSCs, as well as a role in the liver fibrosis.[31] Our *in vivo* data suggest that anti-miR-132 treatment

reversed the inhibition of SIRT1 and caused an overall improvement in liver fibrosis phenotype. Further, our *in vitro* mechanistic data indicated that overexpression of miR-132 reduced, while miR-132 inhibition increased, SIRT1 expression in RAW macrophages, signifying a role for miR-132 in the regulation of SIRT1.

Overall, our data suggest that miR-132 targets multiple genes involved in liver fibrogenesis, and it is likely that the anti-fibrotic phenotype we found in our mouse model is due to the synergistic multitarget effect of miR-132 rather than its effect on a single gene. This is advantageous from a therapeutic point of view for the treatment of complex diseases, including liver fibrosis. In support of our findings, a recent study demonstrated miR-132 as a potential therapeutic target for the treatment of NASH, as anti-miR-132 treatment was effective in ameliorating inflammatory and fibrotic genes and resulted in an overall improvement in steatohepatitis and fibrosis in mouse models of NASH.[57] Dissecting the role of miR-132 at the different stages of liver fibrosis (early, mid, and late) would be the area of growing research in coming years.

In conclusion, the data presented in this study support miR-132 as a crucial player in liver fibrosis. miR-132 was elevated in livers of patients with fibrosis/cirrhosis as well as in a CCl_4-induced liver fibrosis mouse model. Administering LNA-anti-miR-132 attenuated liver fibrosis induced by CCl_4 as well as significantly downregulating inflammatory and pro-fibrotic pathways. We found decreased numbers of EVs and decreased caspase 3 activity after injecting LNA-anti-miR-132 in CCl_4-induced liver fibrosis. We also found association of high levels of miR-132 with SIRT1, and fibrogenic genes with HCC and poor survival outcomes in HCC patients. Collectively, our study suggests a crucial, previously unappreciated role of miR-132 in liver fibrosis. miR-132 could be an interesting candidate with which to develop RNA-RNA interference therapies for the prevention or treatment of liver fibrosis.

MATERIALS AND METHODS

Animal studies

Eight week-old male C57BL/6 wild-type (WT) mice were obtained from Jackson Laboratory (Bar Harbor, ME, USA) and maintained in the animal facility. The study was approved by the University of Massachusetts Medical School Institutional Animal Use and Care Committee (Worcester, MA, USA). Mice were injected intraperitoneally (i.p.) with either miRNA inhibitor scrambled oligonucleotides (LNA-scrambled control) or miRCURY LNA miRNA inhibitor (LNA-anti-miR-132) (15 mg/kg; Exiqon, Woburn, MA, USA) as shown in Figure 2A (n = 8 per group). The number of mice was determined based on previous reports that used *in vivo* delivery of LNA-anti-miRs in other disease models.[58,59] The inhibitors were injected 24 h prior to the CCl_4 regimen every week. Throughout, the experiment, mice received either corn oil (vehicle) or CCl_4 (0.6 mL/kg; i.p. diluted in corn oil at 1:3 ratio) twice a week for a total of 2 weeks, and mice were sacrificed 72 h following the final injection. At the end of treatment, blood was collected from mouse facial veins, and plasma was separated and stored at $-80^{\circ}C$ for further analyses. Liver tissue was immediately either snap frozen in liquid nitrogen for protein analyses or stored in RNAlater (QIAGEN, Valencia, CA, USA) for subsequent RNA analysis.

Some mice were perfused to isolate hepatocytes and KCs using established protocols,[14,60] and one lobe of the liver was dissected out before proceeding for profusion. Briefly, livers were perfused with Hank's balanced salt solution (HBSS) containing EGTA and $CaCl_2$ (buffer 1) for 10 min followed by *in vivo* digestion with HBSS containing collagenase (buffer 2) (Sigma-Aldrich, St. Louis, MO, USA) for 5 min. Liver cells were released from perfused livers in buffer 2 by separating the liver lobes via scalpel under sterile conditions before being filtered through a cell strainer (100 μm). To separate hepatocytes from non-parenchymal cells, the cell suspension was centrifuged at $200 \times g$ for 5 min at room temperature. The pellet containing hepatocytes was washed 2 times with buffer 1 and lysed in Qiazole lysis buffer (QIAGEN, Valencia, CA, USA). To isolate KCs, the supernatant was layered on Percoll gradient (added 25% Percoll in 50 mL tube and underlayer with 50% Percoll) and centrifuged at $1,600 \times g$ for 30 min. The inter-cushion layer was collected carefully and washed with PBS two times, and the resulting cells were cultured in low-glucose Dulbecco's modified Eagle's medium (DMEM) supplemented with 10% fetal bovine serum (FBS) and antibiotics. The free-floating cells were removed after two sessions of 3–4 h of plating with PBS, and new medium was then added. The following day, cells were lysed in Qiazole lysis buffer (QIAGEN, Valencia, CA, USA).

TCGA data analysis

The UALCAN platform was used to analyze HCC tumor transcriptome miR data and plot survival information.[61] *TP53* mutation status was obtained from TCGA whole-exome sequencing data. Mutation Annotation Format (MAF) files (VarScan2) were used for mutation calling. Our analysis included the TCGA liver cancer dataset. The following data are available at https://gdc.cancer.gov/node/977 miR expression and mRNA gene expression. Only samples with both miR expression profiling and mutation or mRNA expression profiling were considered. The specific TCGA dataset applied for this study is provided in Table S1. Correlation between miR-132 and mRNA was quantified with Pearson's correlation coefficient, and correlation coefficients with p value ≤ 0.05 were considered statistically significant. The Mann-Whitney U test was used to compare expression between different groups of samples. We generated survival curves of HCC cases in the TCGA cohort according to the expression status of the miR-132, and the Kaplan-Meier curve was plotted.

Patient samples

Human liver samples were obtained from the National Institutes of Health Liver Tissue Cell Distribution System (Minneapolis, MN, USA). Liver tissues were from control subjects and alcoholic patients with fibrosis/cirrhosis (n = 8).

Histopathological analysis

Formalin-fixed liver sections were stained with Sirius Red stain using standard protocols. The slides were analyzed under light microscopy

at 100× and 200×, and quantification was performed using ImageJ software.

NanoSight/nanoparticle tracking analysis (NTA)

The amounts and diameters of EVs from plasma were determined using the NanoSight NS300 system (NanoSight, UK) as described.[26] NTA post-acquisition settings were kept constant for all samples, and each video was analyzed to give the mean, median, and mode vesicle size as well as concentration estimates. Each sample was measured three times. The concentration of particles (particles/mL) and size distribution (in nanometers) were evaluated using the included NTA software.

Electron microscopy

For electron microscopy, EVs were isolated using the ExoQuick method (System Biosciences, Palo Alto, CA, USA), as described earlier.[62] Briefly, plasma was passed through a 0.8 μm filter, and ExoQuick was added as described by the manufacturer. Purified EVs were re-suspended in PBS then placed on a formvar-coated copper grid and incubated for 30 min as described.[63] The grid was washed with 1× PBS 3 times, and samples were fixed for 10 min by placing the grid onto 2% paraformaldehyde. Fixation was followed by several washes with deionized water, and samples were contrasted by adding 2% uranyl acetate for 15 min. Samples were embedded by adding a drop of 0.13% methyl cellulose and 0.4% uranyl acetate for 10 min before subsequent examination in a Philips CM10 transmission electron microscope and imaging via Gatan CCD digital camera. The purity of EVs was determined by western blot analysis for CD63 expression as described.[41]

Biochemical analysis

Caspase 3 activity was measured from whole liver cell lysates using a caspase 3 activity assay (R&D, Minneapolis, MN, USA), following the manufacturer's suggested protocol.

In vitro transfection studies

RAW 264.7 macrophages were cultured and maintained in high-glucose DMEM (Thermo Fisher Scientific, Waltham, MA, USA) containing 10% FBS (HyClone Laboratories, Logan, UT, USA) at 37°C in a 5% CO_2 atmosphere, as described previously.[50,52]

For overexpression of miR-132, cells were seeded in 24-well plates, and on the next day cells were treated either with a negative control mimic #1 or miR-132 mimic (150 pmol); for inhibition of miR-132, cells were treated either with negative control inhibitor #1 or miR-132 inhibitor (150 pmol) for 24 h (Applied Biosystems, Foster City, CA, USA) using lipofectamine RNAi max reagent (Thermo Fisher Scientific, CA, USA), as described previously.[50,52] Some cells were either treated or not with 0.1% CCl_4 for the last 24 h of the experiment, as described previously.[64] Cells were washed with 1× PBS two times and lysed in RNA lysis buffer for total RNA extraction or radioimmunoprecipitation assay buffer for protein extraction and stored at −80°C for further analysis.

Enzyme-linked immunosorbent assay (ELISA) assay

Cell lysates were used to measure IL-1β protein levels using ELISA as described by manufacturers (BioLegend, San Diego, CA, USA).

RNA analysis and qPCR

10–20 mg of liver tissue was homogenized in QIAzol lysis reagent (QIAGEN, Valencia, CA, USA) using stainless steel beads via TissueLyser II (QIAGEN, Valencia, CA, USA). Total RNA was extracted using the miRNeasy kit (QIAGEN, Valencia, CA, USA) as recommended by the manufacturer.[60] For mRNA analysis, cDNA was transcribed with the iScript reverse transcription system kit (Bio-Rad, Hercules, CA, USA), and quantitative real-time PCR was performed via CFX96 iCycler (Bio-Rad, Hercules, CA, USA). Quantitative analyses of genes were performed using gene-specific primers as presented in Table S2 and as described previously.[65] Cq value was normalized to 18S or β actin mRNA, and differential expression fold changes were calculated using the delta-delta Ct method. For miR analysis, TaqMan miR assays (Applied Biosystems, Foster City, CA, USA) were used as described earlier.[14,60] SnoRNA-202 (mouse samples) or RNU48 (human samples) were used to normalize the technical variations between the samples.

Western blot analysis

Whole-cell lysates were extracted from livers as described.[65] Briefly, 10 mg of liver tissue was homogenized in radioimmunoprecipitation assay buffer containing protease and phosphatase inhibitors via TissueLyser II (QIAGEN, Germany). Homogenized samples were centrifuged, and resulting clear lysates were stored at −80°C. Protein mass was quantified via Bradford assay using Bio-Rad protein assay dye reagent (Bio-Rad, Hercules, CA, USA). Equal amounts of protein (20 μg) were separated in 10% SDS polyacrylamide gel, transferred to nitrocellulose membrane overnight, and blocked for 1 h in blocking buffer. Blot was incubated overnight at 4°C in blocking buffer with primary antibodies for α smooth muscle actin (Abcam cat. no. ab205718) and MMP12 (Abcam, Cambridge, MA, USA), and subsequently washed with 1× Tris-buffered saline with Tween 20 three times. For detection, anti-mouse secondary horseradish peroxidase (HRP)-linked antibodies (Santa Cruz Biotechnology, Dallas, TX, USA) were used for 1 h at room temperature, followed by washing three times with 1× Tris-buffered saline with Tween 20. The immunoreactive bands were detected by chemiluminescence using Pierce ECL western blotting substrate (Pierce Biotechnology, Rockford, IL, USA) and LAS-4000IR (Fujifilm, Valhalla, NY, USA). The same blot was probed with loading control antibody β-actin (Abcam, Cambridge, MA, USA).

Statistical analysis

Statistical significance was determined using the non-parametric Mann-Whitney U test or two-tailed Student's t test for pairwise comparisons based on the underlying data distribution. Non-parametric Kruskal-Wallis or one-way analysis of variance (ANOVA) was used for comparison between more than two groups. Data are presented as mean ± standard error and considered statistically significant at $p < 0.05$.

SUPPLEMENTAL INFORMATION

Supplemental information can be found online at https://doi.org/10.1016/j.omtn.2021.05.007.

ACKNOWLEDGMENTS

The authors thank Dr. Victor Ambros for his valuable suggestions and Dr. Timea Csak for her assistance in animal experiments. This work was supported by startup funds to S.B. by the Department of Medicine, University of Massachusetts Medical School; by grant AA020744 to G.S.; and by grants from the Columbia University Irving Medical Center, the Columbia University College of Dental Medicine, the Irving Institute for Clinical and Translational Research (UL1 TR001873), and Herbert Irving Comprehensive Cancer Center Multi PI grant (P30 CA013696) to F.M.H.

AUTHOR CONTRIBUTIONS

F.M.H.: conceptualization, methodology, validation, investigation, formal analysis, writing, visualization, and funding acquisition. D.C.: investigation. A.T.: writing, review, and editing. G.S.: resources. S.B.: conceptualization, methodology, validation, investigation, formal analysis, visualization, writing, supervision, and funding acquisition.

DECLARATION OF INTERESTS

G.S. reports being a paid consultant for Allergan, Alnylam, Arrow, Durcect Corporation, Generon, Glympse Bio, Terra Firma, Quest Diagnostics, Pandion Therapeutics, Surrozen, and Zomagen. G.S. has received grants from Gilead, Genfit, Intercept, Novartis, SignaBlok, and Shire; she also holds intellectual property rights with UpToDate. All other authors declare no competing interests.

REFERENCES

1. Ambade, A., Satishchandran, A., and Szabo, G. (2016). Alcoholic hepatitis accelerates early hepatobiliary cancer by increasing stemness and miR-122-mediated HIF-1α activation. Sci. Rep. *6*, 21340.
2. Seitz, H.K., Bataller, R., Cortez-Pinto, H., Gao, B., Gual, A., Lackner, C., Mathurin, P., Mueller, S., Szabo, G., and Tsukamoto, H. (2018). Alcoholic liver disease. Nat. Rev. Dis. Primers *4*, 16.
3. Rehm, J., Mathers, C., Popova, S., Thavorncharoensap, M., Teerawattananon, Y., and Patra, J. (2009). Global burden of disease and injury and economic cost attributable to alcohol use and alcohol-use disorders. Lancet *373*, 2223–2233.
4. Shaheen, N.J., Hansen, R.A., Morgan, D.R., Gangarosa, L.M., Ringel, Y., Thiny, M.T., Russo, M.W., and Sandler, R.S. (2006). The burden of gastrointestinal and liver diseases, 2006. Am. J. Gastroenterol. *101*, 2128–2138.
5. Hayashi, H., and Sakai, T. (2011). Animal models for the study of liver fibrosis: new insights from knockout mouse models. Am. J. Physiol. Gastrointest. Liver Physiol. *300*, G729–G738.
6. Friedman, S.L. (2013). Liver fibrosis in 2012: Convergent pathways that cause hepatic fibrosis in NASH. Nat. Rev. Gastroenterol. Hepatol. *10*, 71–72.
7. Kocabayoglu, P., and Friedman, S.L. (2013). Cellular basis of hepatic fibrosis and its role in inflammation and cancer. Front. Biosci. (Schol. Ed.) *5*, 217–230.
8. Ramachandran, P., and Iredale, J.P. (2012). Macrophages: central regulators of hepatic fibrogenesis and fibrosis resolution. J. Hepatol. *56*, 1417–1419.
9. Friedman, S.L., Sheppard, D., Duffield, J.S., and Violette, S. (2013). Therapy for fibrotic diseases: nearing the starting line. Sci. Transl. Med. *5*, 167sr1.
10. Friedman, S.L. (2008). Hepatic stellate cells: protean, multifunctional, and enigmatic cells of the liver. Physiol. Rev. *88*, 125–172.
11. Lee, R.C., Feinbaum, R.L., and Ambros, V. (1993). The C. elegans heterochronic gene lin-4 encodes small RNAs with antisense complementarity to lin-14. Cell *75*, 843–854.
12. Johnson, R., and Buckley, N.J. (2009). Gene dysregulation in Huntington's disease: REST, microRNAs and beyond. Neuromolecular Med. *11*, 183–199.
13. Wanet, A., Tacheny, A., Arnould, T., and Renard, P. (2012). miR-212/132 expression and functions: within and beyond the neuronal compartment. Nucleic Acids Res. *40*, 4742–4753.
14. Bala, S., and Szabo, G. (2012). MicroRNA Signature in Alcoholic Liver Disease. Int. J. Hepatol. *2012*, 498232.
15. Liu, F., Cheng, Z., Li, X., Li, Y., Zhang, H., Li, J., Liu, F., Xu, H., and Li, F. (2017). A Novel Pak1/ATF2/miR-132 Signaling Axis Is Involved in the Hematogenous Metastasis of Gastric Cancer Cells. Mol. Ther. Nucleic Acids *8*, 370–382.
16. Noetel, A., Kwiecinski, M., Elfimova, N., Huang, J., and Odenthal, M. (2012). microRNA are Central Players in Anti- and Profibrotic Gene Regulation during Liver Fibrosis. Front. Physiol. *3*, 49.
17. Tsai, W.C., Hsu, S.D., Hsu, C.S., Lai, T.C., Chen, S.J., Shen, R., Huang, Y., Chen, H.C., Lee, C.H., Tsai, T.F., et al. (2012). MicroRNA-122 plays a critical role in liver homeostasis and hepatocarcinogenesis. J. Clin. Invest. *122*, 2884–2897.
18. He, Y., Huang, C., Zhang, S.P., Sun, X., Long, X.R., and Li, J. (2012). The potential of microRNAs in liver fibrosis. Cell. Signal. *24*, 2268–2272.
19. Strum, J.C., Johnson, J.H., Ward, J., Xie, H., Feild, J., Hester, A., Alford, A., and Waters, K.M. (2009). MicroRNA 132 regulates nutritional stress-induced chemokine production through repression of SirT1. Mol. Endocrinol. *23*, 1876–1884.
20. Alvarez-Saavedra, M., Antoun, G., Yanagiya, A., Oliva-Hernandez, R., Cornejo-Palma, D., Perez-Iratxeta, C., Sonenberg, N., and Cheng, H.Y. (2011). miRNA-132 orchestrates chromatin remodeling and translational control of the circadian clock. Hum. Mol. Genet. *20*, 731–751.
21. Stowell, R.E., Lee, C.S., Tsuboi, K.K., and Villasana, A. (1951). Histochemical and microchemical changes in experimental cirrhosis and hepatoma formation in mice by carbon tetrachloride. Cancer Res. *11*, 345–354.
22. Gao, B., and Bataller, R. (2011). Alcoholic liver disease: pathogenesis and new therapeutic targets. Gastroenterology *141*, 1572–1585.
23. Iredale, J.P. (2007). Models of liver fibrosis: exploring the dynamic nature of inflammation and repair in a solid organ. J. Clin. Invest. *117*, 539–548.
24. Ranghino, A., Dimuccio, V., Papadimitriou, E., and Bussolati, B. (2015). Extracellular vesicles in the urine: markers and mediators of tissue damage and regeneration. Clin. Kidney J. *8*, 23–30.
25. Royo, F., and Falcon-Perez, J.M. (2012). Liver extracellular vesicles in health and disease. J. Extracell. Vesicles. Published online July 11, 2012. https://doi.org/10.3402/jev.v1i0.18825.
26. Momen-Heravi, F., Bala, S., Bukong, T., and Szabo, G. (2014). Exosome-mediated delivery of functionally active miRNA-155 inhibitor to macrophages. Nanomedicine (Lond.) *10*, 1517–1527.
27. Laplante, P., Sirois, I., Raymond, M.A., Kokta, V., Béliveau, A., Prat, A., Pshezhetsky, A.V., and Hébert, M.J. (2010). Caspase-3-mediated secretion of connective tissue growth factor by apoptotic endothelial cells promotes fibrosis. Cell Death Differ. *17*, 291–303.
28. Kakarla, R., Hur, J., Kim, Y.J., Kim, J., and Chwae, Y.J. (2020). Apoptotic cell-derived exosomes: messages from dying cells. Exp. Mol. Med. *52*, 1–6.
29. Manoury, B., Nenan, S., Guenon, I., Boichot, E., Planquois, J.M., Bertrand, C.P., and Lagente, V. (2006). Macrophage metalloelastase (MMP-12) deficiency does not alter bleomycin-induced pulmonary fibrosis in mice. J. Inflamm. (Lond.) *3*, 2.
30. Pellicoro, A., Aucott, R.L., Ramachandran, P., Robson, A.J., Fallowfield, J.A., Snowdon, V.K., Hartland, S.N., Vernon, M., Duffield, J.S., Benyon, R.C., et al. (2012). Elastin accumulation is regulated at the level of degradation by macrophage metalloelastase (MMP-12) during experimental liver fibrosis. Hepatology *55*, 1965–1975.

31. Li, M., Hong, W., Hao, C., Li, L., Wu, D., Shen, A., Lu, J., Zheng, Y., Li, P., and Xu, Y. (2018). SIRT1 antagonizes liver fibrosis by blocking hepatic stellate cell activation in mice. FASEB J. *32*, 500–511.

32. Baffy, G., Brunt, E.M., and Caldwell, S.H. (2012). Hepatocellular carcinoma in non-alcoholic fatty liver disease: an emerging menace. J. Hepatol. *56*, 1384–1391.

33. Lim, Y.S., and Kim, W.R. (2008). The global impact of hepatic fibrosis and end-stage liver disease. Clin. Liver Dis. *12*, 733–746, vii.

34. Chipman, L.B., and Pasquinelli, A.E. (2019). miRNA Targeting: Growing beyond the Seed. Trends Genet. *35*, 215–222.

35. Ucar, A., Vafaizadeh, V., Jarry, H., Fiedler, J., Klemmt, P.A., Thum, T., Groner, B., and Chowdhury, K. (2010). miR-212 and miR-132 are required for epithelial stromal interactions necessary for mouse mammary gland development. Nat. Genet. *42*, 1101–1108.

36. Bijkerk, R., de Bruin, R.G., van Solingen, C., van Gils, J.M., Duijs, J.M., van der Veer, E.P., Rabelink, T.J., Humphreys, B.D., and van Zonneveld, A.J. (2016). Silencing of microRNA-132 reduces renal fibrosis by selectively inhibiting myofibroblast proliferation. Kidney Int. *89*, 1268–1280.

37. Hanin, G., Yayon, N., Tzur, Y., Haviv, R., Bennett, E.R., Udi, S., Krishnamoorthy, Y.R., Kotsiliti, E., Zangen, R., Efron, B., et al. (2018). miRNA-132 induces hepatic steatosis and hyperlipidaemia by synergistic multitarget suppression. Gut *67*, 1124–1134.

38. Chen, Y., Gao, D.Y., and Huang, L. (2015). In vivo delivery of miRNAs for cancer therapy: challenges and strategies. Adv. Drug Deliv. Rev. *81*, 128–141.

39. Stenvang, J., Petri, A., Lindow, M., Obad, S., and Kauppinen, S. (2012). Inhibition of microRNA function by antimiR oligonucleotides. Silence *3*, 1.

40. Petersen, M., and Wengel, J. (2003). LNA: a versatile tool for therapeutics and genomics. Trends Biotechnol. *21*, 74–81.

41. Stenvang, J., and Kauppinen, S. (2008). MicroRNAs as targets for antisense-based therapeutics. Expert Opin. Biol. Ther. *8*, 59–81.

42. Bala, S., Petrasek, J., Mundkur, S., Catalano, D., Levin, I., Ward, J., Alao, H., Kodys, K., and Szabo, G. (2012). Circulating microRNAs in exosomes indicate hepatocyte injury and inflammation in alcoholic, drug-induced, and inflammatory liver diseases. Hepatology *56*, 1946–1957.

43. Momen-Heravi, F., Bala, S., Kodys, K., and Szabo, G. (2015). Exosomes derived from alcohol-treated hepatocytes horizontally transfer liver specific miRNA-122 and sensitize monocytes to LPS. Sci. Rep. *5*, 9991.

44. De Toro, J., Herschlik, L., Waldner, C., and Mongini, C. (2015). Emerging roles of exosomes in normal and pathological conditions: new insights for diagnosis and therapeutic applications. Front. Immunol. *6*, 203.

45. Haviv, R., Oz, E., and Soreq, H. (2018). The Stress-Responding miR-132-3p Shows Evolutionarily Conserved Pathway Interactions. Cell. Mol. Neurobiol. *38*, 141–153.

46. Kucharzewska, P., Christianson, H.C., Welch, J.E., Svensson, K.J., Fredlund, E., Ringnér, M., Mörgelin, M., Bourseau-Guilmain, E., Bengzon, J., and Belting, M. (2013). Exosomes reflect the hypoxic status of glioma cells and mediate hypoxia-dependent activation of vascular cells during tumor development. Proc. Natl. Acad. Sci. USA *110*, 7312–7317.

47. Verma, V.K., Li, H., Wang, R., Hirsova, P., Mushref, M., Liu, Y., Cao, S., Contreras, P.C., Malhi, H., Kamath, P.S., et al. (2016). Alcohol stimulates macrophage activation through caspase-dependent hepatocyte derived release of CD40L containing extracellular vesicles. J. Hepatol. *64*, 651–660.

48. Sirois, I., Raymond, M.A., Brassard, N., Cailhier, J.F., Fedjaev, M., Hamelin, K., Londono, I., Bendayan, M., Pshezhetsky, A.V., and Hébert, M.J. (2011). Caspase-3-dependent export of TCTP: a novel pathway for antiapoptotic intercellular communication. Cell Death Differ. *18*, 549–562.

49. Clark, I.M., Swingler, T.E., Sampieri, C.L., and Edwards, D.R. (2008). The regulation of matrix metalloproteinases and their inhibitors. Int. J. Biochem. Cell Biol. *40*, 1362–1378.

50. Zhang, L., Huang, D., Wang, Q., Shen, D., Wang, Y., Chen, B., Zhang, J., and Gai, L. (2014). MiR-132 inhibits expression of SIRT1 and induces pro-inflammatory processes of vascular endothelial inflammation through blockade of the SREBP-1c metabolic pathway. Cardiovasc. Drugs Ther. *28*, 303–311.

51. Donate, P.B., Alves de Lima, K., Peres, R.S., Almeida, F., Fukada, S.Y., Silva, T.A., Nascimento, D.C., Cecilio, N.T., Talbot, J., Oliveira, R.D., et al. (2021). Cigarette smoke induces *miR-132* in Th17 cells that enhance osteoclastogenesis in inflammatory arthritis. Proc. Natl. Acad. Sci. USA *118*, e2017120118.

52. Chen, I.C., Chiang, W.F., Huang, H.H., Chen, P.F., Shen, Y.Y., and Chiang, H.C. (2014). Role of SIRT1 in regulation of epithelial-to-mesenchymal transition in oral squamous cell carcinoma metastasis. Mol. Cancer *13*, 254.

53. Maubach, G., Lim, M.C., Chen, J., Yang, H., and Zhuo, L. (2011). miRNA studies in in vitro and in vivo activated hepatic stellate cells. World J. Gastroenterol. *17*, 2748–2773.

54. Sabater, L., Locatelli, L., Oakley, F., Hardy, T., French, J., Robinson, S.M., Sen, G., Mann, D.A., and Mann, J. (2020). RNA sequencing reveals changes in the microRNAome of transdifferentiating hepatic stellate cells that are conserved between human and rat. Sci. Rep. *10*, 21708.

55. Zhu, J., Zhang, Z., Zhang, Y., Li, W., Zheng, W., Yu, J., Wang, B., Chen, L., Zhuo, Q., Chen, L., et al. (2018). MicroRNA-212 activates hepatic stellate cells and promotes liver fibrosis via targeting SMAD7. Biochem. Biophys. Res. Commun. *496*, 176–183.

56. Toyoda, H., Tada, T., Yasuda, S., Mizuno, K., Ito, T., and Kumada, T. (2020). Dynamic Evaluation of Liver Fibrosis to Assess the Risk of Hepatocellular Carcinoma in Patients With Chronic Hepatitis C Who Achieved Sustained Virologic Response. Clin. Infect. Dis. *70*, 1208–1214.

57. Papazyan, R., Kinberger, G., Wang, D., Jang, G., Gogas, K., Wright, T., and Zhu, S. (2004). Development of Oligonucleotide-Based miR-132 Antagonists for the Treatment of NASH. Nature *431*, 350–355.

58. Elmén, J., Lindow, M., Schütz, S., Lawrence, M., Petri, A., Obad, S., Lindholm, M., Hedtjärn, M., Hansen, H.F., Berger, U., et al. (2008). LNA-mediated microRNA silencing in non-human primates. Nature *452*, 896–899.

59. Bernardo, B.C., Gao, X.M., Winbanks, C.E., Boey, E.J., Tham, Y.K., Kiriazis, H., Gregorevic, P., Obad, S., Kauppinen, S., Du, X.J., et al. (2012). Therapeutic inhibition of the miR-34 family attenuates pathological cardiac remodeling and improves heart function. Proc. Natl. Acad. Sci. USA *109*, 17615–17620.

60. Bala, S., Marcos, M., Kodys, K., Csak, T., Catalano, D., Mandrekar, P., and Szabo, G. (2011). Up-regulation of microRNA-155 in macrophages contributes to increased tumor necrosis factor alpha (TNFalpha) production via increased mRNA half-life in alcoholic liver disease. J. Biol. Chem. *286*, 1436–1444.

61. Chandrashekar, D.S., Bashel, B., Balasubramanya, S.A.H., Creighton, C.J., Ponce-Rodriguez, I., Chakravarthi, B.V.S.K., and Varambally, S. (2017). UALCAN: A Portal for Facilitating Tumor Subgroup Gene Expression and Survival Analyses. Neoplasia *19*, 649–658.

62. Momen-Heravi, F., and Bala, S. (2018). Extracellular vesicles in oral squamous carcinoma carry oncogenic miRNA profile and reprogram monocytes via NF-κB pathway. Oncotarget *9*, 34838–34854.

63. Momen-Heravi, F., Saha, B., Kodys, K., Catalano, D., Satishchandran, A., and Szabo, G. (2015). Increased number of circulating exosomes and their microRNA cargos are potential novel biomarkers in alcoholic hepatitis. J. Transl. Med. *13*, 261.

64. Bala, S., Calenda, C.D., Catalano, D., Babuta, M., Kodys, K., Nasser, I.A., Vidal, B., and Szabo, G. (2020). Deficiency of miR-208a Exacerbates CCl_4-Induced Acute Liver Injury in Mice by Activating Cell Death Pathways. Hepatol. Commun. *4*, 1487–1501.

65. Bala, S., Csak, T., Saha, B., Zatsiorsky, J., Kodys, K., Catalano, D., Satishchandran, A., and Szabo, G. (2016). The pro-inflammatory effects of miR-155 promote liver fibrosis and alcohol-induced steatohepatitis. J. Hepatol. *64*, 1378–1387.

OMTN, Volume *25*

Supplemental information

Protective effect of LNA-anti-miR-132 therapy on liver fibrosis in mice

Fatemeh Momen-Heravi, Donna Catalano, Austin Talis, Gyongyi Szabo, and Shashi Bala

Materials and Methods

Histopathological Analysis

Formalin-fixed liver sections were stained with hematoxylin-eosin (H&E) using standard protocols. The slides were analyzed under light microscopy at 100X.

Biochemical Analysis

The liver injury was assessed at the enzymatic level by measuring alanine aminotransferase (ALT) activity from plasma samples using a kinetic method (TECO Diagnostics, CA, USA) as per manufacturer's instructions.

Western Blotting

Extracellular vesicles were lysed in RIPA buffer and checked for CD63 expression by Western blot analysis as described previously[1].

Patient Samples

Human liver samples were obtained from the National Institutes of Health Liver Tissue Cell Distribution System (Minneapolis, MN). Liver tissues were from control subjects, and HCV patients with cirrhosis (n=8-10).

Cell Culture

Hepa1.6 mouse hepatocyte cell line was purchased from ATCC and maintained in a low-glucose DMEM (Thermo Fisher Scientific, MA, USA) containing 10% FBS (HyClone Laboratories, UT, USA) at 37°C in a 5% CO_2 atmosphere as described previously[2, 3]. LX2 cells were cultured in low glucose DMEM medium as described previously[4] and treated with 5ng/ml TGFβ for indicated

times. Cells were washed with 1XPBS twice and cells were lysed in Qiazole (Qiagen, USA) and processed for total RNA extraction (Zymo Research, USA).

miR target analysis and correlation studies

Experimentally validated targets of miR-132 in the liver were predicted and visualized by miRNet and correlation studies were performed using TCGA set.

Transfection

For overexpression of miR-132, cells (macrophages and hepatocytes) were seeded onto 24-well plates and next day, cells were treated either with a negative control mimic #1 or miR-132 mimic (150 pmol) and for inhibition of miR-132, cells were treated either with negative control inhibitor #1 or miR-132 inhibitor (150 pmol) for 24h (Applied Biosystems, CA, USA) using lipofectamine RNAi max reagent (Thermo Fisher Scientific, CA, USA) as described previously[2, 3]. Some cells were either treated or not with 0.1% CCl_4 for the last 6h of experiment as described previously[5]. Cells were washed with 1XPBS for two times and lysed in RNA lysis buffer for total RNA extraction or RIPA buffer for protein extraction and stored at -80°C for further analysis.

Electroporation of miRNA into the exosomes and stimulation of RAW macrophages

Loading of control or miR-132 mimic into the exosomes, isolated from THP1 cells, was performed using our previously optimized protocol[1, 6]. Briefly, exosomes were diluted in Gene Pulser® electroporation buffer (Bio-Rad Laboratories, Berkeley, CA) in 1:1 ratio and miR-132 mimic or negative control mimic (Ambion, Grand Island, NY) at 300 pmol were mixed with exosome suspension containing 1 µg/µl exosomal protein. The suspension was transferred into cold 0.2 cm electroporation cuvettes and electroporated at 150 kV and 100 µF using a Gene pulser II System

(Bio-Rad Laboratories, Berkeley, CA) for electroporation. The exosomes were treated with one unit of RNase H to eliminate free-floating miR and re-isolated using ExoQuick-TC™. These loaded exosomes were co-cultured with RAW macrophages for 12 h. Subsequently, cells were washed to remove the free-floating exosomes and cells were cultured in the fresh DMEM high glucose medium for 24h. As a positive control, some cells were treated with LPS (10ng/ml) for 24h. At the end of stimulation, cells were washed with 1XPBS twice and cells were lysed in Qiazole (Qiagen, USA) and processed for total RNA extraction (Zymo Research, USA).

Statistical Analysis

Based on data distribution, one-way analysis of variance (ANOVA) was used to compare different groups. Mann-Whitney *U* test were performed for comparing two groups. Data are presented as mean ± standard error of mean (SEM). P values less than 0.05 was considered as statistically significant.

Supplementary Table 1: TCGA dataset used for analysis.

Supplementary Table 1.xlsx

Supplementary Table 2: Primer sequences are listed below.

**mMMP12**	Forward	TGGCCATTCCTTGGGGCTGC
	Reverse	GGGGGTTTCACTGGGGCTCCATA
**mFoxo3**	Forward	GGAATCGTACGCCCTCCCG
	Reverse	TGCTCTCTCCTCTCGAGCC
**mSIRT1**	Forward	CGGCTACCGAGGTCCATATAC
	Reverse	CAGCTCAGGTGGAGGAATTGT
**mCOX2**	Forward	GCCTACTACAAGTGTTTCTTTTTGCA
	Reverse	CATTTTGTTTGATTGTTCACACCAT
**mIL1B1**	Forward	TGGACCTTCCAGGATGAGGACA
	Reverse	GTTCATCTCGGAGCCTGTAGTG
**mTGFB1**	Forward	CAAGGGCTACCATGCCAACT
	Reverse	GTACTGTGTGTCCAGGCTCCAA
**mMCP1**	Forward	GCTACAAGAGGATCACCAGCAG
	Reverse	GTCTGGACCCATTCCTTCTTGG
**mVimentin**	Forward	CCCTCACCTGTGAAGTGGAT
	Reverse	TCCAGCAGCTTCCTGTAGGT
**mN-cadherin**	Forward	TGAAACGGCGGGATAAAGAG
	Reverse	GGCTCCACAGTATCTGGTTG
**m18s**	Forward	GCAATTATTCCCCATGAACG
	Reverse	GGCCTCACTAAACCATCCAA

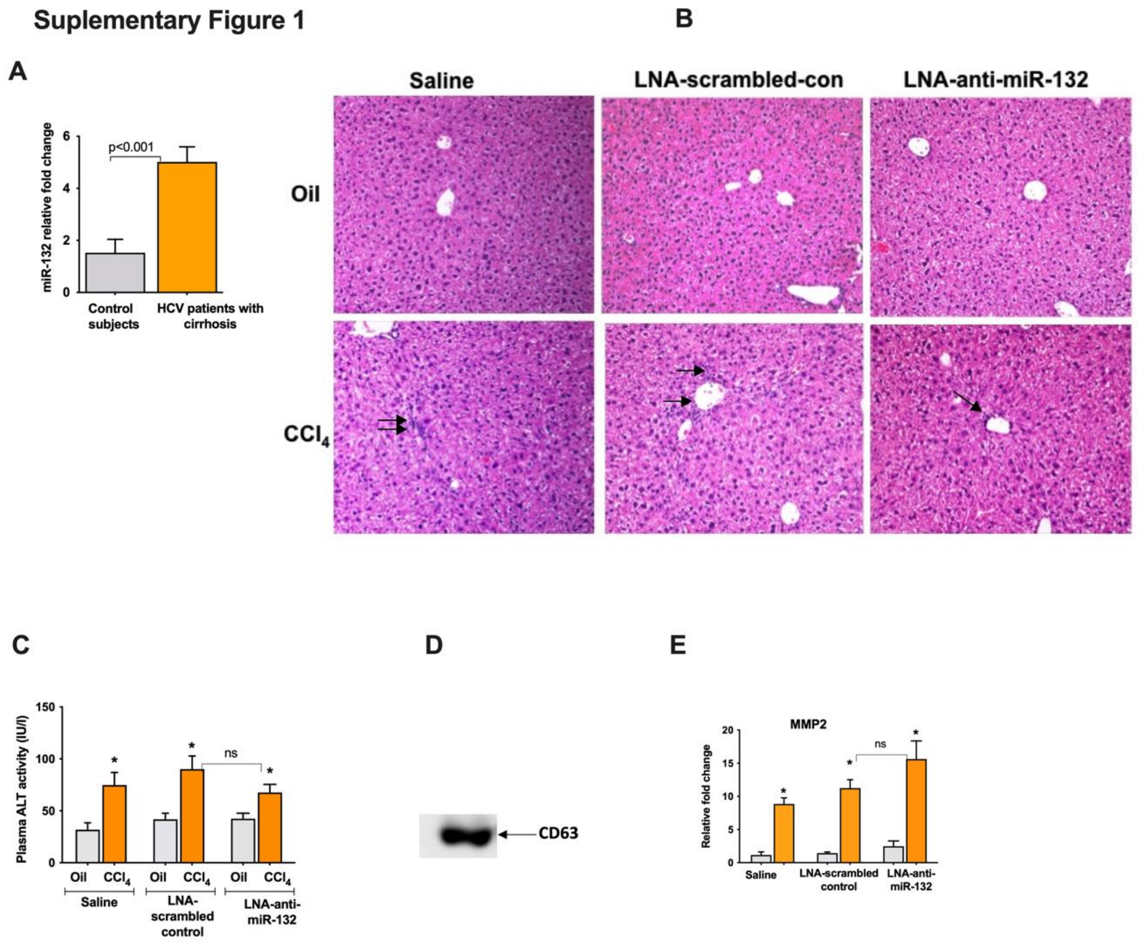

Figure 1. Induction of miR-132 in liver fibrosis/cirrhosis. A) Liver tissues (10mg) of HCV patients with cirrhosis and respective control individuals (n=8-10/group) were used for total RNA extraction using miRNeasy kit. The levels of miR-132 was quantified using TaqMan miR real time PCR assay and RNU48 was used as an internal control. **B)** C57BL/6 male mice (n=8) were injected either with LNA-scrambled control or LNA-anti-miR-132 (15mg/kg) as described in methods. Some mice received either corn oil or CCl_4 (i.p.; 0.6ml/kg of body weight) for indicated times. Representative images of H&E staining of paraffin embedded liver sections. Arrows indicates mononuclear cells. **C)** ALT levels from plasma samples. **D)** CD63 expression from isolated EVs. **E)** MMP2 mRNA transcripts were detected from RNA by real time qPCR and 18S was used to normalize Ct values. Data represent mean ± SEM. Mann-Whitney test or one-way ANOVA was

employed for statistical analysis. * indicates p<0.05 compared to respective control mice. Ns: non significant.

Suplementary Figure 2

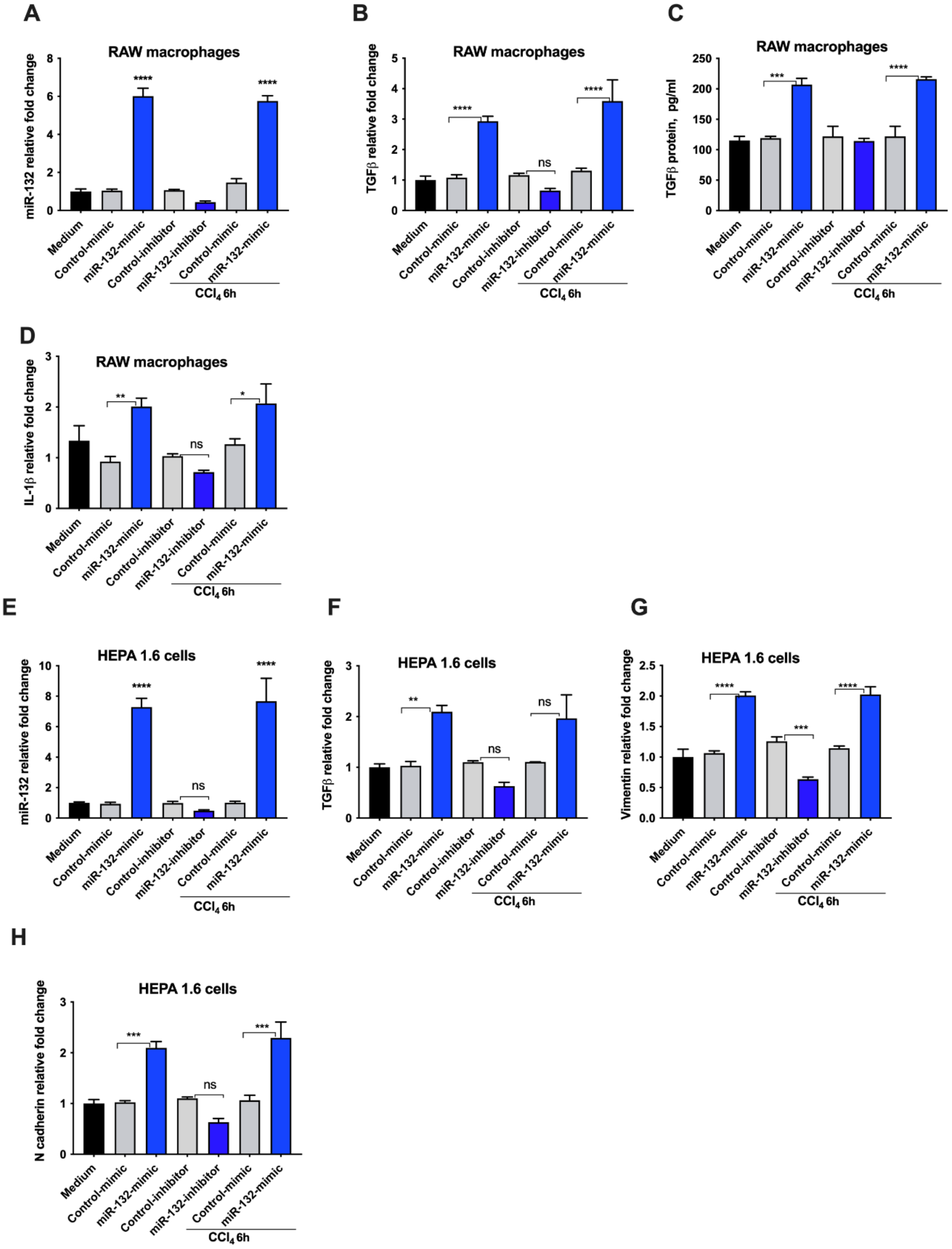

Figure 2. miR-132 regulates the expression of pro-inflammatory and pro-fibrogenic genes. RAW macrophages or Hepa 1.6 hepatocytes were transfected with either control or miR-132 mimic or inhibitor as described in the methods. For the last 6h of transfection, cells were treated or not with 1% CCl_4 and expression of miR-132 (A, E), TGFβ mRNA (B, F) and protein (C) IL-1β mRNA (D) , vimentin (G) and n cadherin (H) was analyzed by real time qPCR and ELISA. Data is shown as mean ± SEM (n=3). Mann-Whitney test or one-way ANOVA was employed for statistical analysis. *, **, ***, **** indicates $p<0.05$, $p<0.005$, $p<0.0005$, $p<0.0001$. ns non-significant.

Suplementary Figure 3

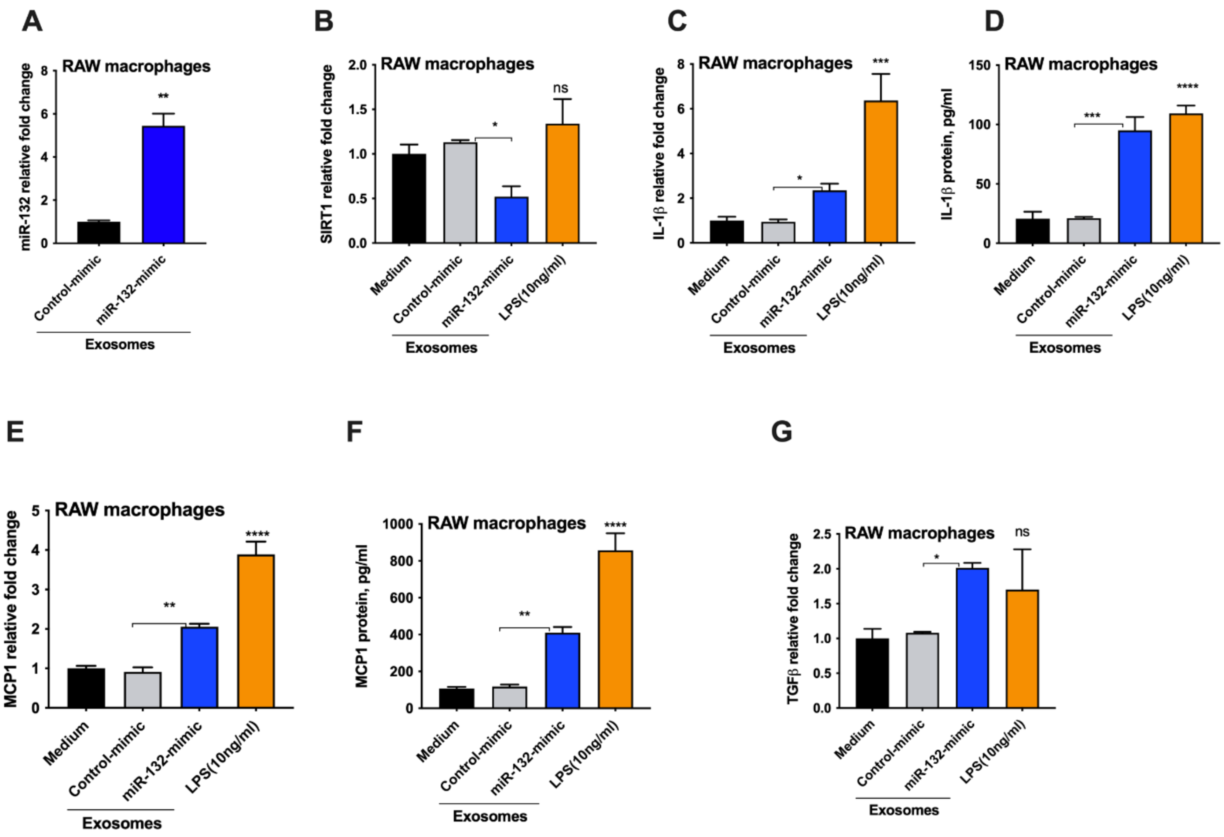

Figure 3. Exosome mediated delivery of miR-132 mimic regulates SIRT1 and inflammatory genes expression in macrophages. Control or miR-132 mimic were loaded into exosomes as

described in the methods. Exosomes were added to naïve RAW macrophages for 12 h and afterwards exosomes were washed off and media was replaced and cultured for 24h. Some cells were treated with10ng/ml LPS for 24h. **A)** miR-132 levels were quantified by real time qPCR and SNORNA-202 was used to normalize Ct values. **B)** SIRT1 **C-D)** IL-1β mRNA and protein levels, **E-F)** MCP1 mRNA and protein levels, and **G)** TGFβ expression was evaluated by real time qPCR and ELISA respectively. 18S was used to normalize Ct values. Data represent mean ± SEM (n=3). one-way ANOVA was employed for statistical analysis. *, **, ***, **** indicates p<0.05, p<0.005, p<0.0005, p<0.0001 respectively compared to control cells. Ns: non-significant.

Suplementary Figure 4

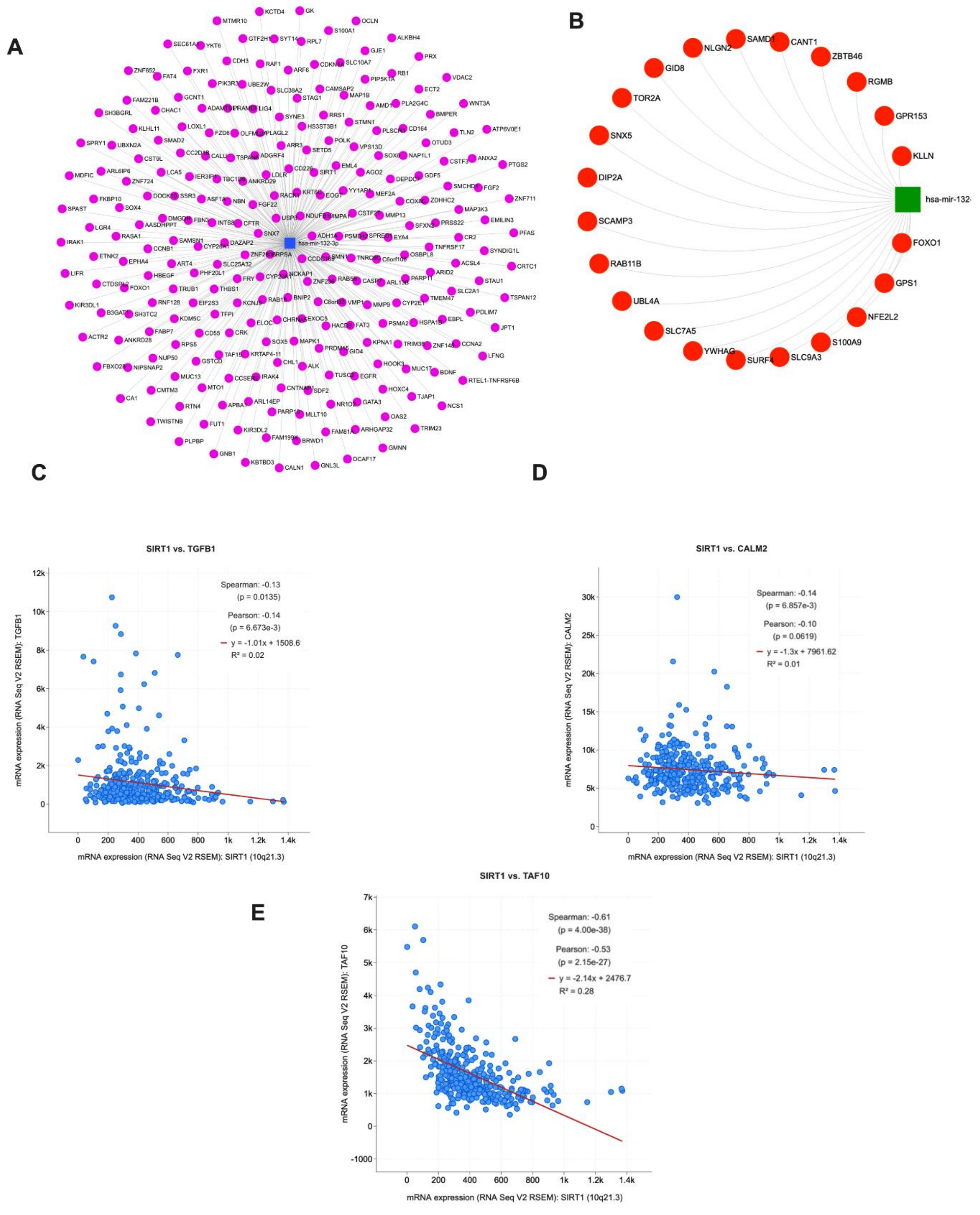

Figure 4. miR-132 target analysis. A) Targets of miR-132 were identified based on experimentally validated miR/mRNA interaction. **B)** Direct hepatocellular carcinoma-related

targets of miR-132 were identified based on experimentally validated miR/mRNA interaction. **C-E)** Correlation between miR-132 target gene SIRT1 was evaluated with fibrogenic markers, TGFβ (C) CALM2 (D) and TAF16 (E).

Supplementary Figure 5

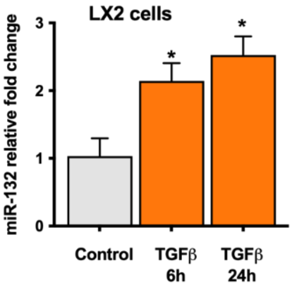

Figure 5. miR expression in hepatic stellate cells. LX2, a human HSC cells were treated with 5ng/ml TGFβ for indicated times and miR-132 levels were quantified and RNU48 was used as an internal control. Data represent mean ± SEM. Mann-Whitney test was employed for statistical analysis. * indicates $p<0.05$ compared to control.

References

1. Momen-Heravi F, Bala S, Kodys K, Szabo G. Exosomes derived from alcohol-treated hepatocytes horizontally transfer liver specific miRNA-122 and sensitize monocytes to LPS. Sci Rep. 2015;5:9991. Epub 2015/05/15. doi: 10.1038/srep09991. PubMed PMID: 25973575; PubMed Central PMCID: PMCPMC4650752.
2. Zhang L, Huang D, Wang Q, Shen D, Wang Y, Chen B, Zhang J, Gai L. MiR-132 inhibits expression of SIRT1 and induces pro-inflammatory processes of vascular endothelial inflammation through blockade of the SREBP-1c metabolic pathway. Cardiovasc Drugs Ther. 2014;28(4):303-11. Epub 2014/06/14. doi: 10.1007/s10557-014-6533-x. PubMed PMID: 24924687.
3. Chen IC, Chiang WF, Huang HH, Chen PF, Shen YY, Chiang HC. Role of SIRT1 in regulation of epithelial-to-mesenchymal transition in oral squamous cell carcinoma metastasis. Mol Cancer. 2014;13:254. Epub 2014/11/27. doi: 10.1186/1476-4598-13-254. PubMed PMID: 25424420; PubMed Central PMCID: PMCPMC4258025.

4. Friedman SL. Hepatic stellate cells: protean, multifunctional, and enigmatic cells of the liver. Physiol Rev. 2008;88(1):125-72. Epub 2008/01/16. doi: 10.1152/physrev.00013.2007. PubMed PMID: 18195085; PubMed Central PMCID: PMCPMC2888531.
5. Bala S, Calenda CD, Catalano D, Babuta M, Kodys K, Nasser IA, Vidal B, Szabo G. Deficiency of miR-208a Exacerbates CCl4-Induced Acute Liver Injury in Mice by Activating Cell Death Pathways. Hepatol Commun. 2020;4(10):1487-501. Epub 2020/10/08. doi: 10.1002/hep4.1540. PubMed PMID: 33024918; PubMed Central PMCID: PMCPMC7527689.
6. Momen-Heravi F, Bala S, Bukong T, Szabo G. Exosome-mediated delivery of functionally active miRNA-155 inhibitor to macrophages. Nanomedicine. 2014;10(7):1517-27. Epub 2014/04/02. doi: 10.1016/j.nano.2014.03.014. PubMed PMID: 24685946; PubMed Central PMCID: PMCPMC4180003.
